# *Toxoplasma gondii* Rhoptry Kinase ROP16 Activates STAT3 and STAT6 Resulting in Cytokine Inhibition and Arginase-1-Dependent Growth Control

**DOI:** 10.1371/journal.ppat.1002236

**Published:** 2011-09-08

**Authors:** Barbara A. Butcher, Barbara A. Fox, Leah M. Rommereim, Sung Guk Kim, Kirk J. Maurer, Felix Yarovinsky, De'Broski R. Herbert, David J. Bzik, Eric Y. Denkers

**Affiliations:** 1 Department of Microbiology and Immunology, College of Veterinary Medicine, Cornell University, Ithaca, New York, United States of America; 2 Department of Microbiology and Immunology, Dartmouth Medical School, Lebanon, New Hampshire, United States of America; 3 Department of Population Medicine and Diagnostic Sciences, College of Veterinary Medicine, Cornell University, Ithaca, New York, United States of America; 4 Center for Animal Resources and Education, College of Veterinary Medicine and Department of Biomedical Sciences, Cornell University, Ithaca, New York, United States of America; 5 Department of Immunology, University of Texas Southwestern Medical Center, Dallas, Texas, United States of America; 6 Division of Immunobiology, Cincinnati Children's Research Foundation, Cincinnati, Ohio, United States of America; University of Pennsylvania, United States of America

## Abstract

The ROP16 kinase of *Toxoplasma gondii* is injected into the host cell cytosol where it activates signal transducer and activator of transcription (STAT)-3 and STAT6. Here, we generated a ROP16 deletion mutant on a Type I parasite strain background, as well as a control complementation mutant with restored ROP16 expression. We investigated the biological role of the ROP16 molecule during *T. gondii* infection. Infection of mouse bone marrow-derived macrophages with *rop16*-deleted (ΔROP16) parasites resulted in increased amounts of IL-12p40 production relative to the ROP16-positive RH parental strain. High level IL-12p40 production in ΔROP16 infection was dependent on the host cell adaptor molecule MyD88, but surprisingly was independent of any previously recognized *T. gondii* triggered pathway linking to MyD88 (TLR2, TLR4, TLR9, TLR11, IL-1ß and IL-18). In addition, ROP16 was found to mediate the suppressive effects of *Toxoplasma* on LPS-induced cytokine synthesis in macrophages and on IFN-γ-induced nitric oxide production by astrocytes and microglial cells. Furthermore, ROP16 triggered synthesis of host cell arginase-1 in a STAT6-dependent manner. In fibroblasts and macrophages, failure to induce arginase-1 by ΔROP16 tachyzoites resulted in resistance to starvation conditions of limiting arginine, an essential amino acid for replication and virulence of this parasite. ΔROP16 tachyzoites that failed to induce host cell arginase-1 displayed increased replication and dissemination during in vivo infection. We conclude that encounter between *Toxoplasma* ROP16 and the host cell STAT signaling cascade has pleiotropic downstream effects that act in multiple and complex ways to direct the course of infection.

## Author Summary

*Toxoplasma gondii* is an extremely widespread intracellular protozoan parasite that establishes long-lasting infection in humans and animals. Because *Toxoplasma* infection is most often asymptomatic, it is evident that this parasite has developed sophisticated ways to manipulate host immunity. Recently, the parasite ROP16 kinase was identified as an important determinant of host cell signaling. During cell invasion, ROP16 is injected into the host cell cytoplasm and subsequently localizes to the nucleus. Here, we report the generation of ROP16 knockout parasites (ΔROP16) as well as ΔROP16 complementation mutants (ΔROP16:1) and we describe the biological effects of deleting and re-inserting this molecule. We find that ROP16 controls the ability to activate multiple host cell signaling pathways and simultaneously suppress macrophage proinflammatory responses. Deletion of ROP16 increases parasite ability to replicate and disseminate during in vivo infection. This increased growth response may arise from ROP16-dependent activation of host arginase-1. Induction of arginase-1 limits availability of arginine, an amino acid that is required for parasite growth and host-inducible nitric oxide production. Our results provide new insight into the complex interactions between an intracellular eukaryotic pathogen and its host cell.

## Introduction

Pathogens living in an intracellular compartment benefit from being able to parasitize their host cell for nutrients, but they are faced with the challenge of surviving within a potentially hostile environment. This is particularly the case for microorganisms that infect cells of innate immunity such as macrophage/monocytes and dendritic cells because these cells possess potent microbial sensing and killing machinery. Many of the mechanisms that viral and bacterial pathogens use to manipulate host cell signaling pathways are well characterized, but less is known about how intracellular eukaryotic pathogens mechanistically interact with host cell signal transduction [1], [2], [3].

For one intracellular protozoan, *Toxoplasma gondii*, this is changing. *Toxoplasma* is an extremely successful microorganism as evidenced by the fact that it infects up to 50% of the human population worldwide and is also a common parasite in domestic and wild animals [4]. Within the host there is evidence that *Toxoplasma* preferentially targets dendritic cells, monocyte/macrophage lineage cells and neutrophils [5], [6], [7]. Infection is normally asymptomatic, and is characterized by widespread dissemination of replicative tachyzoites followed by formation of quiescent cysts in the central nervous system and skeletal muscle tissues that persist for the lifetime of the host with no overt ill effects. Yet, *T. gondii* is an important opportunistic pathogen insofar as infection in immunocompromised patients may have life-threatening consequences, and in utero infection can lead to major defects in the fetus [8], [9]. The parasite is normally controlled by a strong Type 1 cytokine response characterized by high-level production of cytokines such as IL-12p70, TNF-α and IFN-γ [10]. While the latter cytokines are important in resistance to infection, this proinflammatory response must be tightly controlled to avoid immunopathology that can otherwise lead to host death.

It is well established that *Toxoplasma* actively interferes with host cell signaling during intracellular infection of cells such as macrophages (MØ) [11]. The parasite is capable of blocking pathways leading to apoptosis and interfering with proinflammatory responses initiated by signaling through the IFN-γ receptor and Toll-like receptors (TLR). How these suppressive effects occur is less well known, although induction of suppressor of cytokine synthesis (SOCS)-1, activation of phosphatidylinositol (PI)-3 kinase signaling, inhibition of NFκB activation, and blocking chromatin remodeling have each been implicated in parasite-mediated interference with host cell signaling [12], [13], [14], [15], [16].

Recently, the signal transducer and activator of transcription (STAT) signaling pathway has emerged as a major target of exploitation by *T. gondii*. Infection of mouse bone marrow-derived MØ induces rapid and sustained activation of STAT3, a transcription factor through which the anti-inflammatory cytokine IL-10 exerts its function [17]. Additionally, STAT3-negative MØ are much less sensitive to parasite-mediated inhibition of TLR signaling.

Parasite strain type is a critical determinant of STAT3 activation. Of the three predominant strain types, high virulence Type I and low virulence Type III parasites are potent activators of STAT3, whereas low virulence Type II strains are incapable of sustained activation. A major advance in our understanding of how *Toxoplasma* interacts with the STAT signaling machinery came from the results of genetic crosses between *T. gondii* strain types. In these studies the *rop16* locus, contained on chromosome VIIb, emerged as a determinant of activation of both STAT3 and STAT6 [18], [19].

The *rop16* locus encodes rhoptry protein ROP16. This molecule, along with several other proteins, is contained within rhoptries, which are apically associated organelles that discharge their contents during invasion. While many rhoptry proteins localize to the parasitophorous vacuole membrane, some are injected into the host cell cytoplasm associated with empty (e)-vacuoles [20]. The ROP16 molecule is discharged into the cytosol during invasion, and it rapidly translocates to the host cell nucleus via a nuclear translocation sequence [19]. Although originally identified as a putative serine-threonine kinase, recent biochemical studies have shown that ROP16 from Type I parasites is capable of directly catalyzing tyrosine phosphorylation of both STAT3 and STAT6 [21], [22].

Here, we used reverse genetics to generate ROP16 negative (ΔROP16) Type 1 parasites as well as ROP16 complementation mutants (ΔROP16:1), and we studied the biological effects of deleting and re-inserting this gene during in vitro and in vivo infection. We report that ROP16 deletion converts Type I tachyzoites from low to high inducers of IL-12p40, and simultaneously abrogates the suppressive effect of *Toxoplasma* on TLR signaling. We show that ROP16 deletion eliminates the parasite's ability to block nitric oxide production mediated by signaling through the IFN-γ receptor. Yet, paradoxically, ΔROP16 tachyzoites displayed increased replication during in vivo and in vitro infection. We traced this activity to ROP16-dependent STAT6-mediated induction of arginase-1, an enzyme that degrades host cell arginine, which is required for both the production of nitric oxide by host cell inducible nitric oxide synthase, as well as being an essential amino acid nutrient the parasite requires for intracellular replication.

## Results

### An inhibitor of JAK2 blocks parasite-induced STAT3 phosphorylation

Signal transduction resulting in STAT3 tyrosine phosphorylation involves upstream Janus kinase (JAK) activation triggered by cytokine receptor engagement. To determine whether *Toxoplasma* infection stimulates JAK1 or JAK2 phosphorylation, MØ were infected with RH strain tachyzoites, and cell lysates were subjected to immunoprecipitation with anti-phosphotyrosine antibody followed by Western blotting with antibodies specific for JAK1, JAK2 and STAT3 molecules. As shown in Fig. S1A, infection triggered tyrosine phosphorylation of both JAK1 and JAK2, in addition to STAT3.

The embryonic lethality of deleting JAK1 and JAK2 precluded us from examining the role of these molecules in parasite-triggered STAT3 activation, although experiments with *Jak3^−/−^* and *Tyk2^−/−^* MØ revealed that these kinases were not involved (Fig. S1B). However, we found that JAK inhibitor I, a relatively nonspecific reagent, partially blocked parasite-mediated STAT3 activation (Fig. S1C). Furthermore, *Toxoplasma*-induced STAT3 phosphorylation was almost completely blocked by a JAK2-specific inhibitor (Fig. S1D). In contrast, STAT3 phosphorylation stimulated by recombinant IL-6 was largely unaffected by the JAK2 inhibitor. This result is consistent with data suggesting that IL-6-mediated STAT3 activation involves JAK1 and JAK3 molecules [23]. While the most straightforward conclusion of these results is that parasite-triggered JAK2 activation mediates STAT3 phosphorylation, this interpretation must be treated with caution in light of recent evidence that ROP16 directly acts on STAT molecules in a manner sensitive to JAK chemical inhibition [21].

### Generation of ROP16 deletion parasite (ΔROP16) and ROP16 complemented strain (ΔROP16:1)

To examine the biological role of ROP16 regarding STAT activation and modification of host responses, we generated Type 1 parasites lacking expression of this molecule. The ROP16 coding region was targeted and deleted in the Type I RH strain KU80 knockout background [24] (Fig. S2A). PCR assays performed on MPA-resistant clones validated efficient isolation of ROP16 knockouts (ΔROP16) as assessed by deletion of the ROP16 coding region (Fig. S2B), as well as by the correct targeted integration of the *HXGPRT* marker into the deleted ROP16 locus in each MPA-resistant clone (Fig. S2C, D). The ΔROP16 strain was then complemented with a single copy of a functional allele of ROP16 from type I RH by replacement of *HXGPRT* at the ROP16-deleted locus to generate strain ΔROP16:1 (Fig. S2E). The ΔROP16:1 complemented strain exhibited the expected genotype at the ROP16 locus (Fig. S2F, G, H).

### ROP16 deletion attenuates STAT3 tyrosine phosphorylation

**Figure 1:**
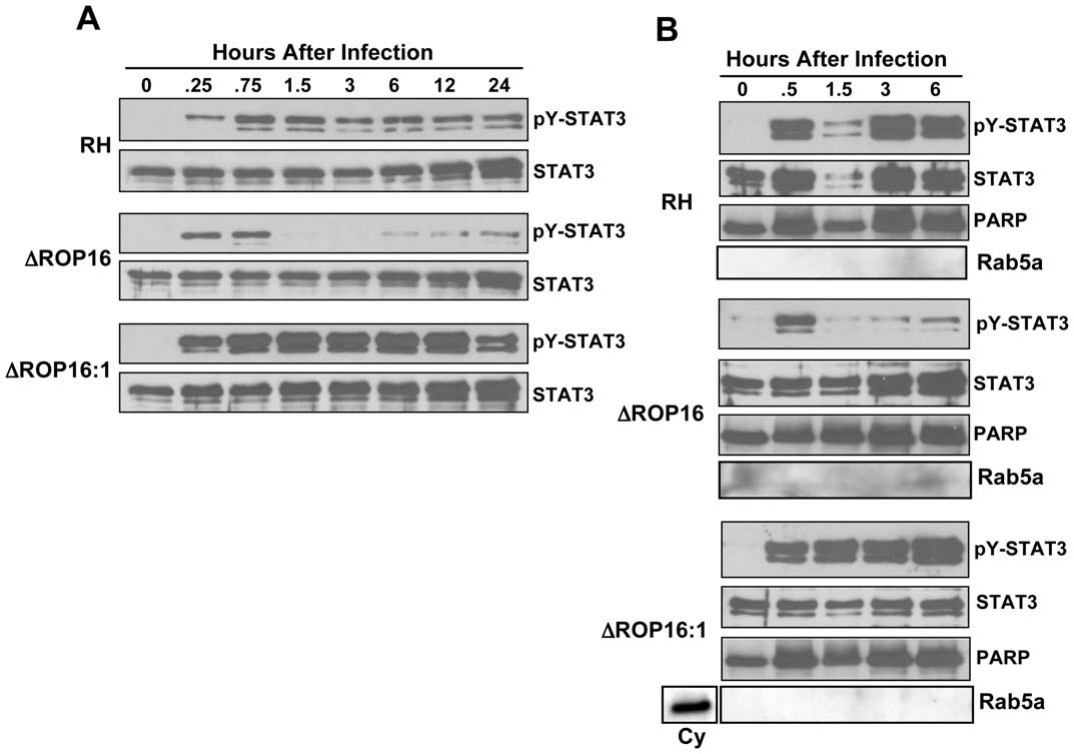
Defective STAT3 tyrosine phosphorylation and nuclear accumulation in the absence of ROP16. (A) Cells were infected with either RH, ΔROP16 or ΔROP16:1 tachyzoites (3∶1 ratio parasites to cells), then total lysates were collected at the indicated times (hr) post-infection and subjected to Western blotting. Blots were probed with anti-phospho-Tyr (Y)^705^ STAT3 then stripped and re-probed with antibody to total STAT3. The experiment was repeated 3 times with similar results. (B) Cells were infected as in (A), but at the indicated times nuclear preparations were prepared and subjected to Western blot analysis for phospho-Tyr^705^ STAT3 and, following membrane stripping, total STAT3. Immunoblotting for PARP and Rab5a, nuclear and cytoplasmic proteins, respectively was also carried out. Cy, control cytoplasmic preparation. This experiment was repeated twice with the same result.

We examined phosphorylation kinetics in MØ infected with either wild-type or ROP16 knockout (ΔROP16) or complemented (ΔROP16:1) parasites (Fig. 1A). As expected, wild-type RH strain tachyzoites induced rapid and sustained STAT3 tyr^705^ (pY-) phosphorylation. In contrast, MØ infected with ΔROP16 parasites displayed early STAT3 tyrosine phosphorylation, but this response was not sustained and phosphorylation levels dropped to background within 1.5 h of infection. Re-introduction of the *rop16* gene into ΔROP16 knockout parasites restored sustained STAT3 activation in the ΔROP16:1 strain (Fig. 1A). Because initial STAT3 phosphorylation is clearly independent of ROP16, but prolonged STAT3 activation requires ROP16, we conclude that activation is a biphasic process mediated initially by a host or parasite kinase independently of ROP16. Yamamoto et al. (22) did not previously report transient ROP16-independent activation of STAT3, most likely because samples were not analyzed prior to 3 hr post-infection. Phosphorylation at the ser^727^ residue is required for full activation of STAT3 [25]. Although this response was more difficult to detect, we found that parasite-mediated ser^727^ STAT3 phosphorylation did not require ROP16 expression (Fig. S3).

Because ROP16 deletion did not completely abrogate STAT3 tyrosine phosphorylation, we examined whether the active transcription factor translocated to the nucleus during infection with ROP16 knockout parasites. Nuclear extracts were prepared from infected MØ and probed for total or pY-STAT3. As demonstrated in Fig. 1B, cells infected with wild-type parasites displayed rapid nuclear accumulation of pY-STAT3. In the absence of ROP16, we also detected rapid accumulation of phosphorylated STAT3 that was greatly reduced by 1.5 hr post-infection even though total STAT3 levels remain elevated. In the complemented ΔROP16:1 strain, levels of phosphorylated STAT3 were maintained in a manner similar to infection with parental parasites. These data are consistent with those of others showing continuous STAT3 shuttling from cytoplasm to nucleus independent of phosphorylation status [26].

### ROP16 down-modulates IL-12p40 production in infected cells

**Figure 2:**
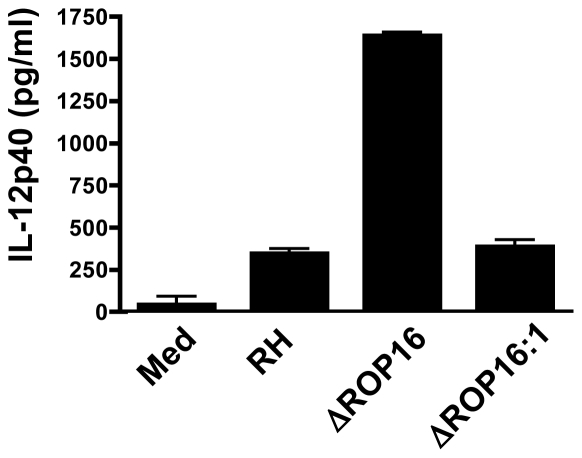
ROP16 negatively regulates IL-12p40 production. Bone marrow-derived MØ were infected at a 3∶1 ratio of parasites to cells, then supernatants were collected after 24 hr and analyzed by ELISA. This experiment was repeated 3 times with similar results.

We, and others, have shown that Type II *Toxoplasma* strains, including PTG and ME49, induce higher levels of the IL-12p40 compared to infection with Type I strains such as RH [27], [28]. Genetic segregation analysis of crosses between parasite strains identified the *rop16* locus as a determinant of IL-12 induction [19]. Therefore, we tested whether deletion of ROP16 would convert the parasite into a strong IL-12 inducer, as would be predicted from the forward genetics studies. As shown in Fig. 2, at 24 hr post-infection parental RH strain tachyzoites induced only low levels of IL-12. In contrast, deletion of ROP16 converted the parasite into a strong IL-12 inducer. Confirming a role for the *rop16* gene in this response, complemented ΔROP16:1 parasites induced low levels of IL-12 similar to wild-type tachyzoites.

**Figure 3:**
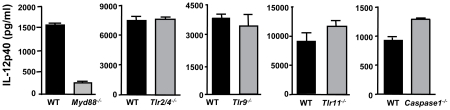
Induction of IL-12 by ΔROP16 tachyzoites requires MyD88, but occurs independently of TLR2, TLR4, TLR9, TLR11, and caspase-1. Bone marrow-derived MØ from wild-type, *Myd88^−/−^, TLR2/4^−/−^*, *TLR9^−/−^*, *TLR11^−/−^* and *caspase-1^−/−^* mice were infected with ΔROP16 tachyzoites (3: 1 ratio of parasites to cells), and supernatants were collected for ELISA 24 hr later. These experiments were repeated twice with similar results.

Because the common adaptor of Toll-like receptor (TLR) signaling, MyD88, has been implicated in immunity to *T. gondii* and in particular in IL-12 MØ responses during Type II infection [29], [30], [31], we examined whether ΔROP16-mediated IL-12p40 production was dependent upon this molecule. As shown in Fig. 3, the ability of ΔROP16 tachyzoites to induce high-level IL-12 production was strongly dependent on MyD88. Previous reports have provided evidence that TLR2 and TLR4 are involved in innate recognition of tachyzoites, suggesting that these receptors might mediate MyD88-dependent IL-12 production that occurs in the absence of ROP16 [32], [33]. However, ΔROP16 parasites induced equivalent levels of IL-12p40 in both wild-type and TLR2/4 double-knockout MØ (Fig. 3). Similarly, TLR9 and TLR11 have been implicated in innate immune recognition of *Toxoplasma* [34], [35], but lack of these molecules had no effect on high-level IL-12 production induced by parasites lacking ROP16 (Fig. 3). Finally, both IL-1β and IL-18 signaling involves MyD88 and it was therefore possible that autocrine production of these cytokines resulted in ΔROP16-mediated IL-12 release. However, MØ lacking caspase-1 (which is required for production of bioactive forms of these cytokines) also produced high amounts of IL-12p40 after infection with ΔROP16 parasites (Fig. 3). In these experiments, littermates served as WT controls and cells were obtained from mice at different facilities, likely contributing to differences on overall cytokine production from strain to strain. Nevertheless, we can conclude that parasite-induced IL-12 occurs independently of these TLR, or alternatively that in the absence of any single TLR, others can functionally substitute.

The MyD88 dependent IL-12 production we observed in the absence of ROP16 recalls previous observations of MyD88 dependent high-level IL-12 production in Type II parasite strain-infected cells [27]. One possibility that would explain the effects of ROP16 on down-regulating IL-12 production is that this rhoptry kinase might control IL-10 production. However, IL-10 was not detected in MyD88*^−^* ^/*−*^ MØ culture supernatants infected with ΔROP16 tachyzoites (data not shown). In addition, we previously demonstrated that IL-10 does not play a role in the inhibition of IL-12 production by WT RH parasites [17].

### Deletion of ROP16 abrogates the ability of *Toxoplasma* to actively inhibit proinflammatory responses

**Figure 4:**
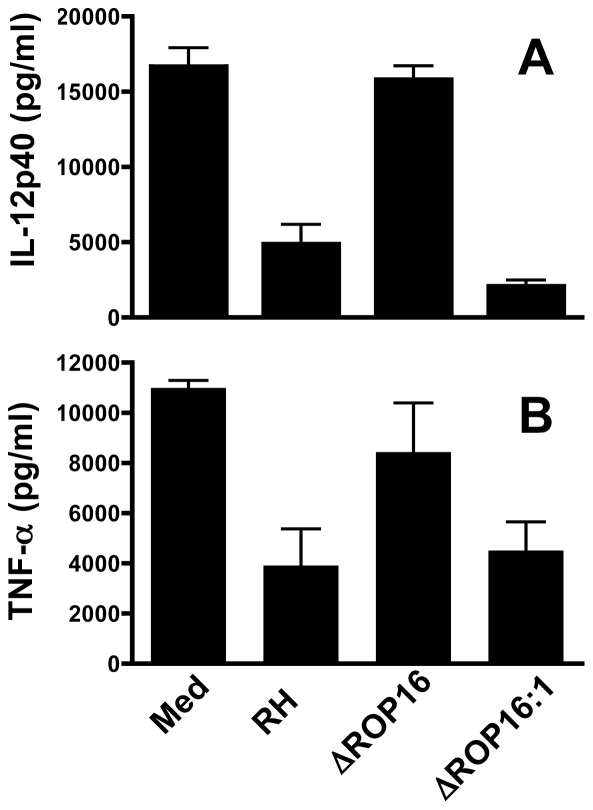
ROP16 controls the ability of *Toxoplasma* to inhibit LPS-induced cytokine production. Bone marrow-derived MØ were infected with RH, ΔROP16 and ΔROP16:1 at a 3∶1 ratio of tachyzoites to cells. After 2 hr, cells were stimulated with LPS (100 ng/ml). Supernatants were collected 6 hr after LPS treatment and analyzed by ELISA for IL-12p40 (A) or TNF-α (B). In these experiments, cytokine levels in supernatants of cells cultured in medium alone or with parasites alone was below the level of detection (50 pg/ml). These experiments were repeated 3 times with similar result.

Previously, we found that infection with Type I RH tachyzoites inhibited MØ responses to LPS and other TLR ligands, and we linked this inhibition phenotype to STAT3 activation [17]. Therefore, we examined whether ROP16 null parasites were defective in their ability to inhibit LPS-induced cytokines. In these experiments, MØ were infected followed 2 hr later by addition of LPS. Six hr after LPS stimulation (at a time prior to induction of IL-12 by parasites themselves) supernatants were collected for ELISA. MØ cultured in medium alone followed by LPS stimulation produced robust amounts of IL-12p40 and TNF-α (Fig. 4A and B, respectively). While cells infected with wild-type RH tachyzoites displayed a defective ability to respond to TLR signaling, ROP16-deleted parasites lost their ability to suppress LPS-induced cytokine production. However, re-introduction of the *rop16* gene restored the parasites' ability to strongly suppress LPS-induced IL-12p40 and TNF-α production (Fig. 4A and B). Similar inhibitory effects of the RH strain on TLR-dependent stimulation occur during infection of DC [5], [36]. In contrast, ROP16 negative parasites failed to display this strong inhibitory effect during infection of DC (data not shown).

**Figure 5:**
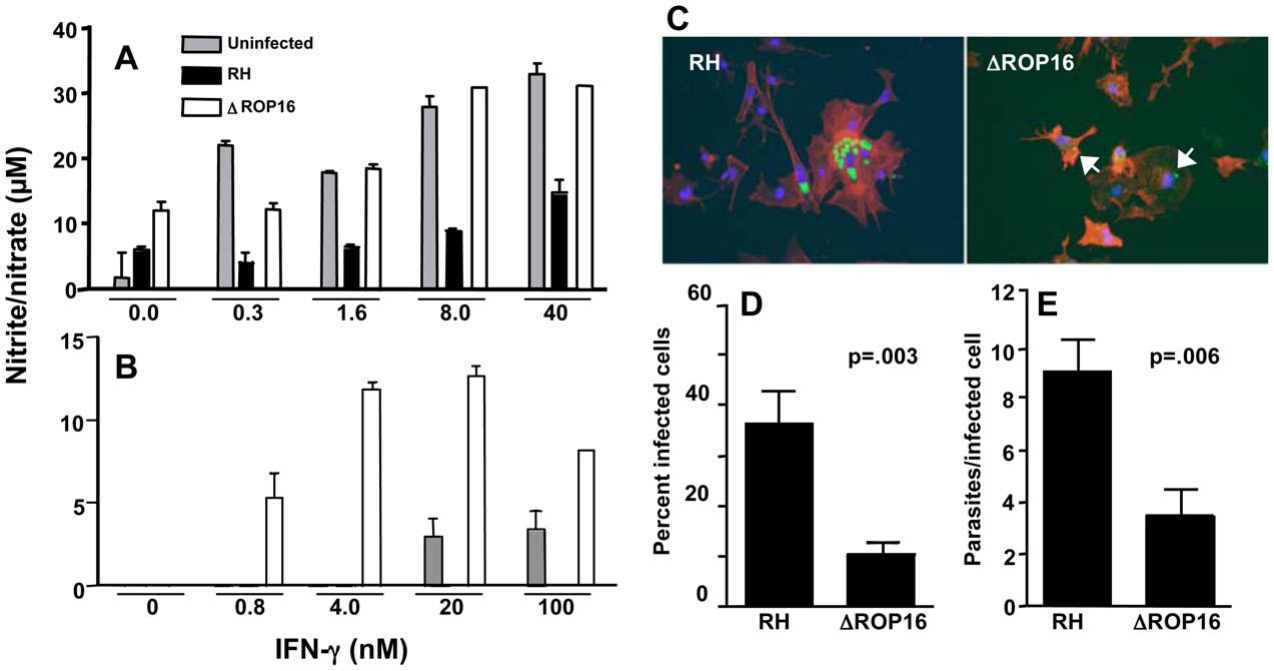
ΔROP16 tachyzoites do not inhibit IFN-γ primed NO production in microglial cells and ΔROP16 parasites potentiate production in astrocytes. Neonatal microglial cells (A) or astrocytes (B) were preincubated with the indicated amounts of IFN-γ for 3 hr and either left uninfected (grey bars) or infected with RH (black bars) or ΔROP16 (white bars) parasites (3∶1 ratio of tachyzoites to cells). After 48 hr, supernatants were analyzed for nitrite/nitrate as production as a measure of NO. (C) RH tachyzoites survive and replicate in IFN-γ activated astrocytes, but ΔROP16 parasites are killed. Cultures were primed for 3 hr with IFN-γ, infected with parasites (at a ratio of 1∶1) and cultured an additional 48 hr before immunofluorescence assay. Arrowheads point to degraded ΔROP16 parasites that stain irregularly with Ab specific for tachyzoite surface antigen (SAG)-1. Green, parasites; red, phalloidin-stained actin; blue, nucleus. Both percentage of infected cells (D) and number of parasites per infected cell (E) are significantly higher in RH- versus ΔROP16-infected cultures. Micrographs were scored for percent infection and intracellular parasite count. At least 10 fields and 100 cells were counted for each infection. These collective experiments were repeated twice with the same result.

In addition to inhibition of signaling mediated through TLR pathways, *Toxoplasma* is capable of down-regulating IFN-γ-triggered signaling that leads to iNOS-dependent nitric oxide (NO) production [37]. Stimulation with IFN-γ alone failed to elicit significant amounts of NO in bone marrow-derived MØ, thioglycollate elicited MØ, or bone marrow-derived DC **(**data not shown). In addition, none of the parasite strains by themselves triggered iNOS or NO production. However, both microglial cells and astrocytes from noninfected neonatal mice produced NO after IFN-γ stimulation. When microglial cells were infected with RH tachyzoites and subjected to cytokine stimulation, NO production was severely limited (Fig. 5A). In marked contrast, infection with ROP16 null parasites failed to result in NO inhibition. IFN-γ stimulation of astrocytes resulted in lower amounts of NO, and this response was blocked during RH infection (Fig. 5B). Interestingly, when these cells were infected with ΔROP16 tachyzoites, IFN-γ-dependent NO production was even greater than that detected after stimulation with IFN-γ alone. Indeed, at priming concentrations of 4 nM and lower, IFN-γ failed to elicit detectable NO unless cells were infected with ΔROP16 parasites (Fig. 5B). Taken together, we conclude that ROP16 is a major molecule mediating inhibition of downstream targets of both TLR and IFN-γ receptor signaling pathways.

Because ΔROP16 parasites did not block and even potentiated NO production by astrocytes, and because NO is important in controlling the parasite in the brain, we examined tachyzoite survival in astrocyte-enriched cell cultures. Cells were subjected to priming with IFN-γ for 3 hr and then infected with RH or ΔROP16 parasites for 48 hr. Both parasite strains infected cells equally well and replicated in the absence of IFN-γ (data not shown). However, as shown in Fig. 5C, while RH survived and replicated within IFN-γ primed astrocytes, ΔROP16 parasites were significantly more susceptible to killing. ΔROP16 parasites displayed weak labeling with Ab to tachyzoite-specific surface antigen (SAG)-1 (p30) and the parasite form was compromised (arrows in right panel of Fig. 5C). The percent infection (Fig. 5D) and the number of tachyzoites per cell (Fig. 5E) were significantly less in ΔROP16-infected astrocytes compared to RH-infected cells.

### ROP16 mediates STAT6-dependent arginase-1 induction

**Figure 6:**
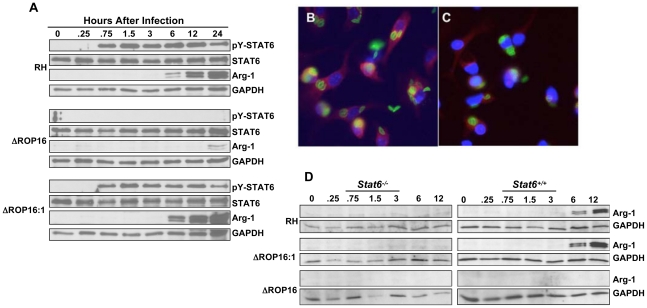
ROP16 controls STAT6-dependent arginase-1 synthesis. (A) Bone marrow-derived MØ were infected with RH, ΔROP16 and ΔROP16:1 tachyzoites (3: 1 ratio of parasites to cells), then at the indicated times (hr) post-infection total cell lysates were subjected to Western blotting with phospho-STAT6 antibody. Blots were successively stripped and re-probed with antibodies to total STAT6, arginase-1 (Arg-1) and GAPDH. (B and C) Bone marrow-derived MØ were infected with RH (B) or ΔROP16 (C), then 24 hr later cells were fixed and subjected to immunofluorescence staining for arginase-1 expression. Red, arginase-1; green, *Toxoplasma*; blue, nucleus. (D) *Stat6^−/−^* and *Stat6^+/+^* bone marrow-derived MØ were infected with the indicated parasite strains, then lysed and subjected to Western blot analysis with anti-Arg-1 antibody. Blots were subsequently stripped and re-probed with a GAPDH-specific antibody. These experiments were performed 3 times with the same result.

In addition to STAT3 activation, genetic linkage studies implicated the *rop16* locus in activation of STAT6. A major downstream target of STAT6 signaling is arginase-1. This signaling cascade was of interest because arginase-1 competes with iNOS for the amino acid substrate arginine [38], [39], [40]. Therefore, we examined whether ROP16 could stimulate STAT6-dependent arginase-1 production. As shown in Fig. 6A, RH induced strong and sustained tyr^641^ phosphorylation of STAT6. In marked contrast, this response was completely absent in ΔROP16 mutant tachyzoites. However, in the *rop16* complemented strain, STAT6 activation was restored (Fig. 6A). The absolute dependence of STAT6 phosphorylation on ROP16 differed from STAT3 activation, where only early ROP16-independent phosphorylation occurred (Fig. 1A and Fig. S4, which shows nuclear lysates from a single experiment blotted and probed for STAT3, then re-probed for STAT6). Interestingly, we detected nuclear accumulation of total STAT6, despite lack of detectable STAT6 tyrosine phosphorylation during ΔROP16 infection (Fig. S4). This may indicate phosphorylation-independent STAT nuclear accumulation as reported by others [41].

Paralleling the STAT6 phosphorylation results, wild-type and complemented parasites, but not ΔROP16 tachyzoites, induced arginase-1 synthesis, a response detectable by immunoblotting for arginase-1 protein within 6 hr of infection (Fig. 6A). Similarly, we detected induction of arginase-1 by immunofluorescence assay during RH but not ΔROP16 infection (Fig. 6B and C, respectively). Because the *arginase-1* gene is controlled by STAT6 signaling, we examined whether parasite-induced induction of arginase-1 also depended upon STAT6. As shown in Fig. 6D, infection of STAT6*^−^* ^/*−*^ MØ with all three strains failed to induce arginase-1 protein, in contrast to infection of MØ from STAT6^+/+^ littermates in which we detected ROP16-dependent arginase-1 induction. We did not detect production of IL-4/IL-13 in these cultures making it very unlikely that arginase-1 induction was due to ROP16-dependent production of these cytokines (data not shown).

### ROP16 modulates the availability of the essential amino acid arginine

**Figure 7:**
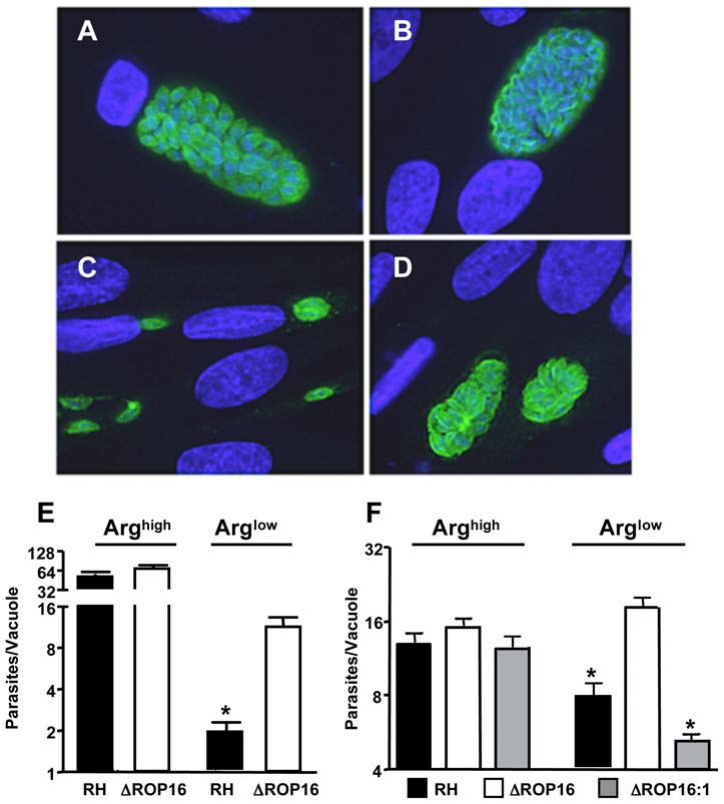
ΔROP16 parasites are resistant to arginine deficient conditions. (A-D) Fibroblasts were cultured in complete (A, B) or arginine-deficient (C, D) medium 24 hr prior to and during infection with RH (A, C) or ΔROP16 (B, D) tachyzoites. Cells were fixed and stained for intracellular parasites (green) 48 hr after infection. Nuclear staining (DAPI) is shown in blue. (E) Number of tachyzoites per vacuole in infected fibroblasts under normal and arginine-deficient conditions. (F) The experiments were repeated in bone marrow-derived MØ using RH, ΔROP16 and ΔROP16:1 tachyzoites at a 1∶1 ratio of parasites to cells. * p<0.05. These experiments were repeated three times with similar result.

*Toxoplasma* is known to be an arginine auxotroph, strictly relying on either host or exogenously supplied arginine for its replication and survival [42]. ROP16-dependent induction of arginase-1 suggested that parasites lacking this rhoptry molecule might be more resistant to conditions of arginine limitation. Therefore, we compared the ability of wild type and ΔROP16 parasites to invade and multiply within fibroblasts cultured in arginine-replete and arginine-deficient media. In normal arginine-replete medium, RH (Fig. 7A) and ΔROP16 (Fig. 7B) parasites displayed vigorous multiplication rates (quantitated in Fig. 7E). However, under arginine limiting conditions, RH replication was severely curtailed and only one cycle of replication occurred during the 38 hr growth assay (Fig. 7C and E). In striking contrast, replication of ΔROP16 parasites was markedly less affected by arginine starvation, with evidence of 4 or more cycles of division (Fig. 7D and E).

Although *Toxoplasma* is known as a microorganism that can infect virtually any kind of nucleated cell, the parasite is often found preferentially in macrophage/DC lineage cells during in vivo infection [7]. Therefore, we also examined the growth behavior of RH, ΔROP16 and ΔROP16:1 parasites under arginine high and low conditions in bone marrow-derived MØ. As shown in Fig. 7F, while all three strains replicated equivalently under arginine replete conditions, deletion of ROP16 resulted in a growth advantage to the parasites when arginine was present in limiting concentration.

**Figure 8:**
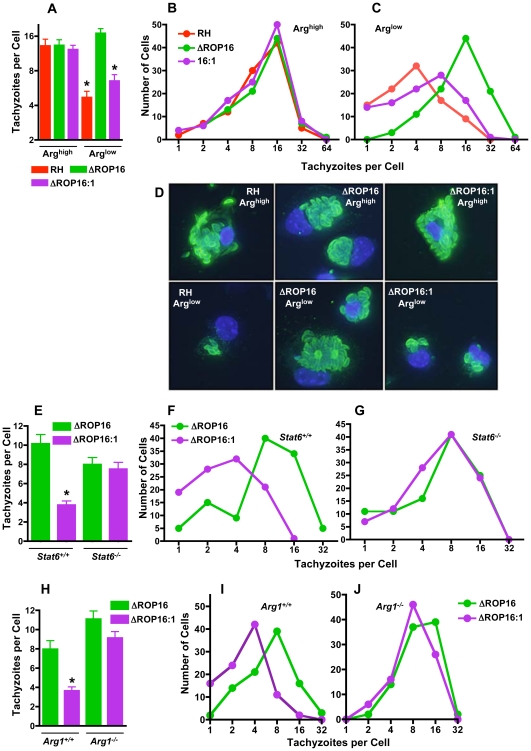
ROP16 mediates resistance to arginine limitation during ex vivo infection of peritoneal exudate cells. Resident peritoneal cells were collected, cultured under Arg^high^ or Arg^low^ conditions and infected with the three parasite strains for 48 hr. In (A), the average number of tachyzoites per cell was calculated for approximately 100 infected cells. In B and C, the number of cells harboring the indicated number of parasites is shown, depicting the downshift in replication cycles for RH and the ROP16-complemented strains when arginine is limited (C). (D) Representative images of infected peritoneal exudate cells for experiments in A-C. In panel E, the average number of tachyzoites per cell (n = 100 infected cells) is shown 48 hr after infection of resident macrophages from *Stat6^+/+^* and *Stat6^−/−^* mice. Number of cells containing the indicated number of parasites is shown for *Stat6^+/+^* (F) and *Stat6^−/−^* (G) resident peritoneal MØ. Panel H, number of tachyzoites per cell (n = 100) at 48 hr post-infection in *Arg1^−/−^* and *Arg1^+/+^* MØ. Panels I and J show number of parasites per cell in *Arg1^+/+^* and *Arg1^−/−^* MØ, respectively. *, p<0.05.

Along similar lines, we collected mouse resident peritoneal cells, which are composed largely of CD11b^+^ resting MØ and carried out ex vivo infection with RH, ΔROP16 and ΔROP16:1 parasites under arginine high and low conditions. As in mouse bone marrow-derived MØ and human fibroblasts, in arginine limiting conditions deletion of ROP16 conferred a significant growth advantage on parasites as measured by overall average numbers of tachyzoites per cell (Fig. 8A). While the distribution of parasite number per cell was similar for all strains under arginine high conditions (Fig. 8B), the presence of ROP16 in RH or the complemented ΔROP16:1 strain reduced the number of parasites per cell under arginine limiting conditions (Fig. 8C). In contrast, the ROP16-deleted strain exhibited significantly higher numbers of parasites per cell (Fig. 8C). Representative image of cells infected with each mutant under arginine high and low conditions are shown in Fig. 8D.

The above results argued that ROP16/STAT6-dependent arginase-1 induction played a role on slowing parasite growth. To test this, we compared growth of ΔROP16 and ΔROP16:1 parasites in peritoneal macrophages from *Stat6^+/+^* and *Stat6^−/−^* mice. Parasites expressing Type I ROP16 clearly displayed a growth disadvantage in WT cells that was eliminated in *Stat6* gene-deleted macrophages (Fig. 8E-G). As expected ΔROP16 parasites, which do not trigger STAT6 activation, replicated equivalently in wild-type and knockout cells (Fig. 8E–G). Next, we performed the same experiment employing macrophages from *Arg1^−/−^* and *Arg1^+/+^* littermate control mice. As shown in Fig. 8H-J, while ΔROP16:1 replicated less well than ΔROP16 parasites in wild-type MØ, the strains replicated equivalently in *Arg1^−/−^* cells. We conclude that ROP16 mediates STAT6-dependent arginase-1 induction, thereby modulating arginine availability and resulting in parasite sensitivity to arginine starvation conditions.

### Influence of ROP16 on IL-12 production, parasite replication, and dissemination during in vivo infection

**Figure 9:**
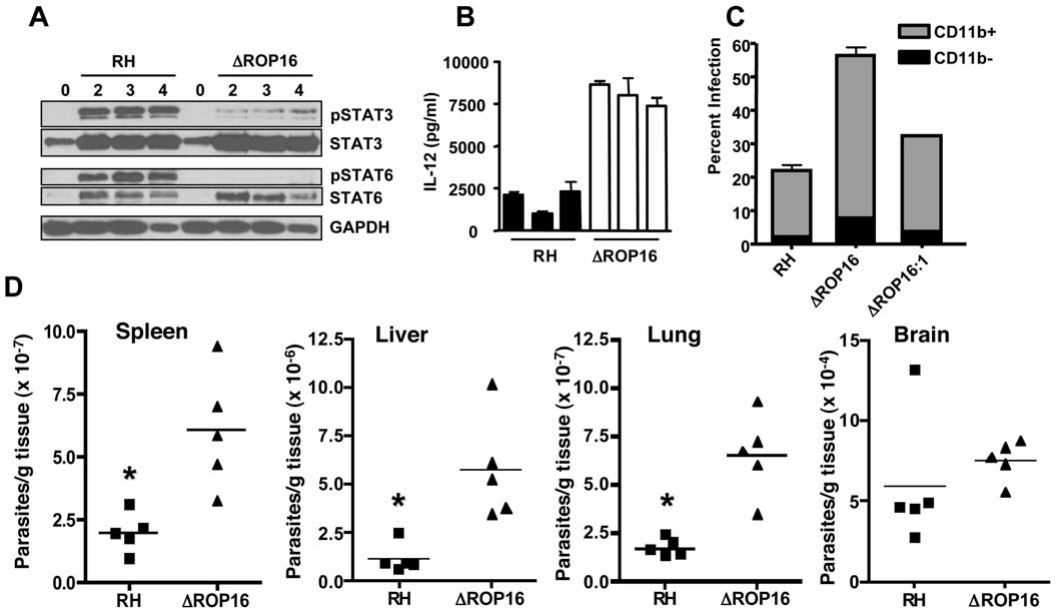
Effects of ROP16 on cytokine production and parasite replication during in vivo infection. (A) C57BL/6 mice were infected by i. p. injection with 10^6^ tachyzoites of either RH or ΔROP16 strains. Peritoneal exudate cells were collected at 2, 3 and 4 days post-infection, lysed and subjected to Western blot analysis using antibodies to total and phosphorylated STAT3 and STAT6. (B) Peritoneal exudate cells collected 24 hr post-infection were cultured 24 hr without further stimulation and supernatants were assayed for IL-12p40 cytokine. Bars represent individual mice. (C) Mice (n = 4 per group) were infected with 10^5^ RH, ΔROP16, ΔROP16:1 tachyzoites, then cells were collected 48 hr later and stained for surface CD11b expression and intracellular tachyzoites using anti-SAG-1 antibody followed by flow cytometric analysis. The data show percent infected cells in CD11b^+^ and CD11b^−^ populations. D, Mice were i.p. infected with 10^6^ tachyzoites and organs were harvest 72 hr post infection. Tissues were subjected to quantitative real time PCR amplification of the *Toxoplasma* B1 gene. Each symbol represents a single mouse. *p<0.01. This experiment was performed twice with similar results.

To investigate the effect of ROP16 deletion during in vivo infection, mice were i. p. inoculated with wild-type, or ΔROP16 parasites. Peritoneal exudate cells (PEC) were collected 2, 3 and 4 days post-infection and analyzed by Western blot for STAT3 and STAT6 phosphorylation. In agreement with our in vitro results, both of these signaling intermediates were phosphorylated in a ROP16-dependent manner (Fig. 9A). In Fig. S5, we i. p. infected mice with RH, ΔROP16 and ΔROP16:1 and collected PEC 5 days later. The results confirm that deletion and re-introduction of ROP16 controls STAT3 and STAT6 activation in vivo. PEC collected 24 hr post-infection were analyzed for ex vivo IL-12 production. As shown in Fig. 9B, cells from ΔROP16-infected mice produced significantly higher levels of IL-12p40 relative to cells from mice infected with wild-type parasites. Both wild-type and ΔROP16-infected mice succumbed with the same kinetics after i. p. infection, despite evidence for increased IL-12 production during ΔROP16 infection (Fig. S6).

We examined the course of acute infection in the peritoneal cavity following parasite inoculation with 10^6^ tachyzoites. At 6 hr post infection (Fig. S7), similar levels of CD11b^+^ cells were observed in the RH and ΔROP16 infections, and the percent infection was equivalent. Flow cytometric analysis revealed the CD11b^+^ cells to be inflammatory monocytes and approximately 10% neutrophils (data not shown). By 24 hr post-infection, the percentage of infected cells was approximately 2-fold higher in mice infected with ΔROP16 parasites. After 72 hr of infection, 80% of PECs were infected with ROP16 knockout parasites compared to 54% with the wild-type parasite strain. We also infected mice with a 10-fold lower dose of parasites and collected PEC 72 hr later for parasite quantitation by qPCR. The results (Fig. S7B) show increased numbers of parasites per host cell during infection with ΔROP16 relative to WT parasites.

**Figure 10:**
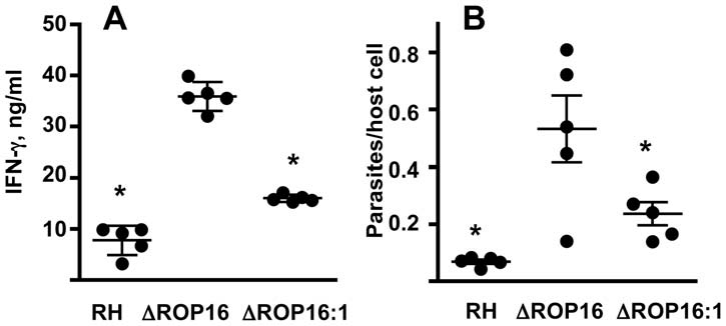
ΔROP16 infection induces high-level IFN-γ production in spleen. (A) Splenocytes were isolated from Day 7 mice infected with each of the three strains (10^5^ tachyzoites) and cultured without further stimulation for 72h. Supernatants were assayed for IFN-γ. (B) Splenocytes were also subjected to qPCR amplification of the *Toxoplasma* B1 gene and parasites were related to cell number by determining genome equivalents of host argininosuccinate lyase. *, p<0.02.

We performed similar experiments employing ΔROP16:1 tachyzoites in parallel with RH and ΔROP16 parasites. As shown in Fig. 9C, while deletion of *rop16* promoted parasite infection in the peritoneal cavity, re-introduction of the gene into the deletion mutant decreased tachyzoite-positive cells to levels similar to RH infection. Increased parasite level resulting from ROP16 deletion was also found in various tissues (liver, lung, spleen) harvested from 3-day infected mice. Levels of the parasite B1 gene were significantly higher after infection with ΔROP16 parasites relative to the parental RH strain in all tissues except for the brain (Fig. 9D). In Fig. 10, we assessed downstream IFN-γ production and parasite numbers in spleen following infection. In the spleen IFN-γ levels displayed a ROP16-dependent increase (Fig. 10A). There was a concomitant increase in parasites per host cell in the spleen that depended upon the presence of Type I ROP16 (Fig. 10B).

### Evidence that ROP16-dependent arginase-1 induction in infected MØ limits in vivo parasite replication and dissemination

**Figure 11:**
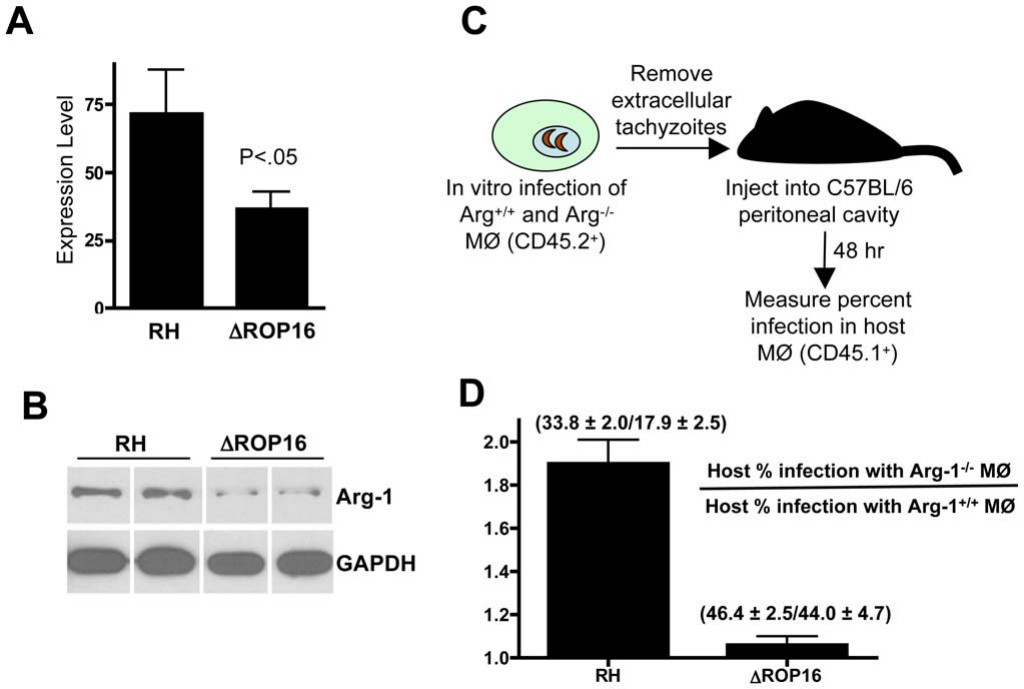
ROP16-dependent arginase-1 induction limits in vivo infection. (A) C57BL/6 mice (3 per group) were infected with the indicated parasite strains and qPCR for the *Arg-1* gene was performed on brain RNA at 4 days post-infection. Expression levels are shown relative to levels in noninfected mice. (B) Mice were infected with RH or ΔROP16 tachyzoites then 4 days later mesenteric lymph node lysates were subjected to immunoblot analysis using antibodies to arginase-1 and GAPDH. Each band represents the result from an individual animal. (C) Schematic for transfer of infected *Arg-1^+/+^* or *Arg-1^−/−^* CD45.2^+^ MØ into congenic CD45.1^+^ host peritoneal cavities. (D) Relative infection level in host cells following transfer of RH or ΔROP16-infected *Arg-1^+/+^* and *Arg1^−/−^* MØ. Infection in host CD45.2^+^ cells was measured 48 hr following transfer of infected MØ. The data are plotted as host infection in recipients of infected *Arg1^−/−^* relative to *Arg1^+/+^* MØ, and the numbers indicate percent infection ± SD in each case. These experiments were repeated 2–3 times with similar results.

We next examined whether ROP16 was involved in arginase-1 induction during in vivo infection. To test this, we infected mice by i. p. injection with 10^6^ parasites of either strain and measured *Arg-1* expression levels by qPCR. Within 4 days of infection, we detected strong *Arg-1* up regulation during RH infection. Notably, RH induced approximately two-fold more *Arg-1* than ΔROP16 (Fig. 11A). Likewise, when mesenteric lymph node lysates were subjected to Western blotting for arginase-1 (Fig. 11B), we found that the protein was expressed at higher level during RH infection.

We tested the hypothesis that ROP16-mediated arginase-1 expression in infected cells limited the replication and dissemination of the parasite during in vivo infection. Straightforward infection of arginase-1 knockout or MØ-specific arginase-1 knockout mice was problematic because of issues of lethality for arginase-1 knockout mice. In addition, cytokines such as IL-4 produced during infection could mediate arginase-1 induction in infected and noninfected cells, and this could also complicate the results. Therefore, we adopted the approach outlined in Fig. 11C. Wild-type and *Arg1^−/−^* CD45.1^+^ MØ were infected with RH or with ΔROP16 parasites. After removing extracellular tachyzoites, infected MØ were transferred into the peritoneal cavities of wild-type CD45.2^+^ congenic mice. Two days after transfer, PEC were collected and host cell infection levels were analyzed by flow cytometry. We reasoned that if ROP16-mediated arginase-1 induction limited in vivo growth, emergence of ΔROP16 parasites and subsequent infection of CD45.2 host cells would occur more rapidly than during RH infection. The results are plotted as the ratio of percent host infection in recipients of infected *Arg1^−/−^* MØ relative to recipients of *Arg1^+/+^* MØ. Indeed, there was large increase in infection rate when wild-type parasites entered the host in *Arg1^−/−^* MØ compared to *Arg1^+/+^* cells (Fig. 11D). In contrast, the MØ genotype made no difference in dissemination to the host during ΔROP16 infection.

## Discussion

*Toxoplasma* displays an unusual population structure in that 3 clonal lineages predominate in Europe and North America [43]. Type I strains are highly virulent in mice, whereas strain types II and III are less virulent and can establish latent infection. Importantly, there is evidence that Type I strains also cause more serious disease in humans [44]. Studies in mice suggest that the immune response is an important determinant of virulence, although its exact role is unclear. For example, infection with Type I strains is associated with overproduction of proinflammatory cytokines, whereas the response during Type II infection is more restrained [45], [46]. Paradoxically, Type II tachyzoites stimulate higher levels of MØ IL-12 production compared to Type I parasites during in vitro infection [27], [28].

*Toxoplasma* strain type is an important determinant of activation of STAT signaling pathways during intracellular infection. Forward genetic analysis identified ROP16 as a polymorphic parasite kinase controlling strain-specific activation of STAT3 and STAT6, as well as modulating production of IL-12p40 [19]. Here, we employed a reverse genetic approach to generate ROP16 deletion mutants as well as control complementation mutants to gain insight into the biological role of this rhoptry molecule during infection.

Previously, it was found that Type I strain parasites induces rapid and sustained STAT3 activation associated with low-level IL-12 production in mouse MØ [17], [27], [28]. In contrast, Type II *Toxoplasma* fails to sustain STAT3 activation, and this was genetically linked to high level IL-12 synthesis [19]. We found Type I parasites lacking ROP16 were severely defective in STAT3 tyrosine phosphorylation. Nevertheless, ΔROP16 parasites maintained an ability to trigger early STAT3 activation. This result is essentially identical to previous data from genetic crosses indicating that Type I ROP16 is required for sustained STAT3 activation rather than the initial response [19]. Nevertheless, recent in vitro kinase studies have shown that both STAT3 and STAT6 serve as direct substrates for ROP16 tyrosine kinase activity [21], [22]. Therefore, we propose that there are two STAT3 activation phases. The first occurs rapidly and independently of ROP16, and possibly involves activation of JAK molecules. The second wave is necessary for sustained STAT3 activation in *Toxoplasma*-infected cells and is likely dependent upon direct tyrosine kinase activity of Type I ROP16. Interestingly, STAT6 tyrosine phosphorylation differed from STAT3 tyrosine phosphorylation insofar as deletion of ROP16 completely eliminated the parasite's ability to activate this signal transducing molecule. Therefore, STAT6 activation during infection is entirely dependent upon the parasite kinase.

Deletion of ROP16 converts Type I parasites from low to high inducers of IL-12. Here, we show that this response, like that induced by the Type II *Toxoplasma* ME49 strain, is highly dependent upon the common adaptor MyD88. This molecule is involved in signal transduction through most TLR, as well as signaling through receptors for IL-1β and IL-18 [47]. We assessed whether TLR2, 4, 9 and 11, MyD88-dependent TLR implicated in the response to *Toxoplasma* [48], were responsible for high level IL-12 production induced by the ΔROP16 strain. However, using TLR knockout MØ, we found no evidence for involvement of these TLR, and we formally ruled out autocrine IL-1β and IL-18 activity using MØ from caspase-1 knockout mice. The ROP16-dependent and MyD88-dependent IL-12 production that we observe may result from redundant functions of multiple TLR, or it is possible that *Toxoplasma* itself may be capable of bypassing TLR and use a novel mechanism to directly trigger MyD88-dependent signaling.

*Toxoplasma* is known to inhibit signaling through TLR ligands such as LPS, and we previously found that this activity was dependent upon STAT3 [17]. Here, we show that ROP16 controls the ability to suppress TLR4-triggered IL-12p40 and TNF-α production. We reported recently that *T. gondii* interferes with LPS-induced chromatin remodeling at the TNF promoter by blocking phosphorylation and acetylation of histone H3, suggesting one mechanism for the suppressive effects of the parasite [49]. However, ROP16 does not appear to be involved in this activity, because ΔROP16 parasites maintain the ability to inhibit TLR4-mediated histone H3 modification (data not shown). The biological significance of the down-regulatory effects of *Toxoplasma* on TLR signaling is not yet clear. Since *Toxoplasma* infection is naturally acquired via oral ingestion of parasites, one possibility is that inhibition is a way to evade the activating effects of bacterial TLR ligands that the host is exposed to during *T. gondii* infection in the intestine [50], [51]. Alternatively, it is possible that down-regulating TLR signaling is a way for the parasite to avoid the activating effects of its own TLR ligands.

*Toxoplasma* also interferes with signaling mediated by IFN-γ [37], [52], [53]. In order to assess this response, we first examined bone marrow-derived and thioglycollate-elicited MØ NO production. In our hands these cells produced undetectable amounts of NO in response to IFN-γ (data not shown). However, both astrocytes and microglial cells are known to produce NO [54], [55], and indeed we found that IFN-γ stimulation of these cells resulted in NO release. While wild-type parasites were able to suppress the response, the ΔROP16 strain was defective in inhibitory activity. Insight into the functional significance of this response may come from the observation that ability to express inducible NO, in particular by microglial cells, is involved in controlling chronic infection in the mouse brain, whereas animals survive acute infection without the iNOS enzyme [56], [57], [58]. Therefore, expression of ROP16 may be a parasite mechanism to increase transmission potential by escaping the microbicidal effects of NO in the central nervous system.

Despite the finding that absence of ROP16 results in enhanced IL-12 production and defective ability to inhibit production of proinflammatory mediators, replication and dissemination of tachyzoites was enhanced by deletion of ROP16. We obtained evidence that the reduced replication and dissemination of parental RH parasites compared to ΔROP16 parasites was due to arginine starvation resulting from ROP16-dependent STAT6-mediated induction of arginase-1. The dependence of *Toxoplasma*-induced arginase-1 expression on STAT6 activation we observed is in contrast to previously published results by El Kasmi et al. [40]. In that study, the authors observed STAT6 activation by Type II ROP16, a result not seen by us or others [19], [21]. Likewise, the authors reported STAT6-independent arginase-1 induction during infection with Type II ROP16-expressing parasites, a result that also stands in contrast to our findings. At present we do not understand the reason for these discrepant results, but because El Kasmi et al. used ME49 without manipulation of ROP16 a certain degree of caution regarding STAT6-dependent arginase-1 induction may be warranted.

Regardless, our data are consistent with a view that ROP16-mediated induction of arginase-1 functions to limit parasite replication, and that this is a strategy to facilitate host survival and establishment of latent infection to increase transmission potential. An alternative view comes from the consideration that arginase-1 and iNOS compete for the same substrate - namely, arginine. Thus, ROP16-mediated arginase-1 induction, and consequent arginine depletion in infected cells, may represent a mechanism used by Type I strain parasites to evade the potentially lethal effects of high-level NO production. Consistent with this concept, we previously reported that Type II ME49 infection, in contrast to Type I RH infection, resulted in NO production during in vivo infection in the spleen even though overall cytokine synthesis was greater during Type I infection [45], [46].

More evidence for an in vivo role of arginase-1 during infection comes from recent findings with *Leishmania major* and *Schistosoma mansoni* [59], [60]. In those studies, arginase-1 induction in myeloid cells promoted infection by localized depletion of arginine, in turn leading to suppressed T cell responses. This could possibly have relevance to in vivo responses during *Toxoplasma* infection, insofar as several older studies suggested that acute infection is associated with nonspecific T cell suppression [61], [62], [63]. We are currently re-examining this issue.

Our study demonstrates ROP16 manipulates host cell signaling pathways that determine availability of arginine for parasite replication and dissemination, and host production of NO. By manipulating arginase-1 levels and consequently arginine availability to both host and parasite, ROP16 may act as a central regulator of parasite replication and transmission potential. In addition to ROP16-mediated control of host arginase-1, it seems possible, and even likely, that ROP16 has additional functions during intracellular infection. The ROP16 molecule is involved in activation of STAT3 and STAT6, transcription factors that each possess their own unique targets. Microarray analysis of host cell responses also suggests that ROP16 has multiple downstream targets [21]. Thus, ROP16 is emerging as a molecule at the nexus of the host-parasite interaction, and as such may function as one of the key determinants of strain-specific virulence and transmissibility.

While this manuscript was under review similar findings were reported by an independent group (Jensen et al. Cell Host and Microbe 2011. 9:472).

## Materials and Methods

### Ethics statement

The experiments in this study were performed in strict accordance with the recommendations in the Guide for the Care and Use of Laboratory Animals of the National Institutes of Health. The protocols were approved by the Institutional Animal Care and Use Committee at Cornell University (permit number 1995-0057). All efforts were made to minimize animal suffering during the course of these studies.

### Mice

Female C57BL/6 mice, 6–8 wks of age, were purchased from Taconic Farms (Germantown, NY). *STAT6^−/−^* mice (provided by M. Bynoe), *TLR2^−/−^/TLR4^−/−^* mice (provided by D. Russell) and *MyD88^−/−^* animals, originally supplied by S. Akira (Osaka University), were bred in-house at Cornell University. *Caspase-1^−/−^* and *JAK3^−/−^* mice were obtained from The Jackson Laboratory (Bar Harbor, ME). *TLR9^−/−^* mice were bred in house at UT-Southwestern Medical School and *Arg-1^−^* ^/*−*^ mice were bred at the University of Cincinnati College of Medicine. Animals were housed under specific pathogen-free conditions in the Cornell University College of Veterinary Medicine animal facility, which is accredited by the Association for the Assessment and Accreditation of Laboratory Animal Care International.

### Parasites

Tachyzoites were maintained by twice weekly passage on human foreskin fibroblast monolayers in DMEM (Life Technologies, Gaithersburg, MD) supplemented with 1% heat-inactivated bovine growth serum (HyClone, Logan, UT), 100 U/ml penicillin (Life Technologies), and 0.1 mg/ml streptomycin (Life Technologies). Parasite cultures were tested every 4–6 weeks for *Mycoplasma* contamination using a commercial PCR-ELISA based kit (Roche Applied Systems, Mannheim, Germany).

### Antibody sources

Cell Signaling Technology (Danvers, MA) was the source of antibodies to total and phospho-STAT3, JAK1, JAK2, ERK1/2 PARP and GAPDH. Anti-CD11b and anti-phospho-STAT6 were purchased from BD Biosciences (San Jose, CA). FITC-conjugated anti-p30 was purchased from Argene, Inc. Dr. Sidney Morris (University of Pittsburgh) generously provided the arginase-1 antibody.

### Gene knockout targeting plasmid constructs

The Type I ROP16 gene locus is defined by TGGT1_063760 in the current *T. gondii* genome database www.Toxodb.org (version 6.0). Gene knockout targeting plasmid pΔROP16 was constructed by fusing through yeast recombinational cloning [64] a ∼1.2 kb 5′ ROP16 target flank amplified from RH genomic DNA, the *HXGPRT* minigene cassette [24] and a ∼1.2 kb 3′ ROP16 target flank amplified from RH genomic DNA in correct order into the yeast-shuttle plasmid pRS416. The deletion was engineered to remove a small portion of the 5′ UTR and essentially all of the coding region with the exception of a few codons near the predicted translation stop of ROP16, to create a ∼2.2 kb deletion in the ΔROP16 knockout strain. Complementation plasmid pΔROP16:1 was generated by fusing a c-terminal HA-tagged functional allele of Type I RH ROP16 with 5′ and 3′ ROP16 target flanks. The oligonucleotide primers used in gene knockout and complementation plasmid construction are shown in Table S1. Targeting plasmids were validated by restriction digest, and by DNA sequencing to verify 100% homology in gene targeting flanks.

### Gene replacement at the *rop16* locus

Approximately 10 µg of PmeI linearized pΔROP16 targeting plasmid was individually transfected into *T. gondii* strain RHΔ*ku80*Δ*hxgprt* that exhibits highly enhanced homologous recombination and knockouts were then selected in mycophenolic acid (MPA) using previously described methods [24]. Individual MPA resistant clones were isolated and the genotype of the clones was evaluated by PCR as previously described [24]. Genotype validation primers are shown in Table S1. PCR 1 (*rop16* deletion) used primers ΔF and ΔR. PCR 2 used primers EXF & CXR. PCR 3 (5′ integration) used primers CXF and pminiHXR. PCR 4 (3′ integration) used primers CXR and pminiHXF [24]. The ΔROP16:1 complemented strain was generated by retargeting the ROP16-deleted locus with plasmid pΔROP16:1 to delete *HXGPRT* and reinsert a c-terminal HA-tagged functional allele of Type I RH ROP16. Following transfection of the ΔROP16 knockout strain with 10 µg of PmeI linearized pΔROP16:1 targeting plasmid, parasites were selected in 6-thioxanthine (6TX) for removal of the *HXGPRT* selectable marker [24]. The complemented ΔROP16:1 strain was validated in PCR by showing correct 5′ and 3′ integration of the Type I ROP16 allele at the ROP16 locus. PCR 5 (5′ integration) used primers CXF and cvR. PCR 6 (3′ integration) used primers cvF and CXR.

### Virulence assay

Adult 8-week-old female CF1 mice were obtained from Charles River Laboratories and maintained in Techniplast Seal Safe mouse cages on vent racks at the Dartmouth-Hitchcock Medical Center (Lebanon, NH) mouse facility. All mice were cared for and handled according to the Animal Care and Use Program of Dartmouth College using National Institutes of Health-approved institutional animal care and use committee guidelines. Groups of four mice were injected intraperitoneally with 0.2 ml (100 tachyzoites) and monitored daily for degree of illness and survival.

### Bone marrow-derived MØ and DC preparation

MØ were derived from bone marrow by 5-day culture in L929-containing supernatants as previously described [65]. DC were prepared as described previously [66].

### Generation of astrocytes and microglia

Astrocytes and microglia were generated from the brains of 3-4 day old mice as previously described [67] with minor modifications. Briefly, brains were homogenized through 40-micron cell strainers and single cell suspensions were subjected to discontinuous Percoll gradient centrifugation (70∶30∶0). Astrocytes were collected from the 30∶0 interface and microglial cells from the 70∶30 interface. Both cell types were cultured in DMEM/F12 medium supplemented with 10% bovine growth serum up to three weeks. Microglial cells were additionally supplemented with 130 ng/ml recombinant GM-CSF (PeproTech, Inc. Rocky Hill NJ). Medium was replaced every 3 days.

### Cell culture

Infection was accomplished by addition of tachyzoites to cell cultures (3∶1 ratio of parasites to MØ). Plates were briefly centrifuged (3 min, 200 x *g*) to synchronize contact between tachyzoites and cells. For endotoxin triggering studies, LPS (100 ng/ml; *S. minnesota*, ultrapure, List Biological Laboratories, Campbell, CA) was added 60 min after infection, and at varying times cells were collected for biochemical assays. For measurement of cytokine release, cells were infected with various parasite ratios with and without subsequent LPS stimulation. Supernatants were collected at times indicated in text. Total cell lysates and nuclear extracts were prepared for Western blot analysis with the Nuclear Extract Kit (Active Motif, Carlsbad, CA). In some experiments, JAK inhibitor I (EMD Biosciences, La Jolla, CA) and JAK2 inhibitor III (EMD Biosciences) were added to cells 60 min prior to infection. Arginine-free medium (Sigma-Aldrich) was supplemented with dialyzed bovine growth serum, leucine, lysine and glucose. In addition, arginine-high medium was supplemented to 84 mg/L L-arginine. Arginine-low medium was not supplemented with the amino acid.

### Immunoblot analysis

Cells (10^6^/sample) were lysed in reducing SDS sample buffer, and DNA was sheared by forcing samples three times through a 27-gauge needle. After boiling for 3 min, cellular lysates were separated by 10% SDS-PAGE, and proteins subsequently electrotransferred onto nitrocellulose membranes (Schleicher & Schuell, Keene, NH). Membranes were blocked in 5% nonfat dry milk containing 0.1% Tween 20 (Sigma-Aldrich) in Tris-buffered saline, pH 7.6 (TBST), for 1 hr at room temperature, followed by incubation with primary antibodies according to the manufacturer's protocol (Cell Signaling Technology). After washing blots in TBST, antibody binding was detected with an HRP-conjugated secondary antibody (Jackson ImmunoResearch Laboratories, West Grove, PA) in TBST containing 5% nonfat dry milk for 1 hr at room temperature. Tyrosine phosphorylated STAT6 was detected using a mouse antibody from BD Biosciences followed by anti-mouse biotin-conjugated antibody (Thermo Scientific, Rockford IL) and an anti-biotin horseradish peroxidase-conjugated antibody (Cell Signaling Technology). After washing blots in TBST, bands were visualized using an ECL system (Lumi-GLO; Cell Signaling Technology).

### Pull-down assay

Tachyzoites were added to bone marrow-derived MØ (5×10^7^) at a ratio of 8: 1, cells were pelleted (2000 rpm, 3 min), and incubated for 20 min at 37°C. In some experiments, recombinant IL-6 (eBioscience; San Diego, CA; 100 ng/ml) was added. Cells were washed in PBS and resuspended in Cell Lysis Buffer containing a cocktail of protease and phosphates inhibitors (Cell Signaling Technology), and the suspension was subjected to brief sonication. After centrifugation (14, 000 rpm, 10 min, 4°C), the resulting pellet was resuspended in 100 µl anti-phosphotyrosine-agarose (Cell Signaling Technology) and the slurry was rotated overnight at 4°C. The agarose beads were subsequently washed in Cell Lysis Buffer, resuspended in SDS sample reducing buffer and incubated at 100°C for 3 min. After pelleting the beads, samples were subject to SDS-PAGE and immunoblot analysis.

### Immunofluorescence microscopy

Coverslips bearing infected MØ or fibroblast monolayers were fixed with 4% formaldehyde in PBS, rinsed with PBS and incubated (1 h, room temperature) with indicated primary antibodies and/or FITC-conjugated anti-P30 antibody diluted in PBS containing 1% BSA and 10% donkey serum. Coverslips were washed 3 times with PBS and then incubated (1 h, room temperature) with secondary antibody in the same diluent. After washing 3 times with PBS, coverslips were mounted with Pro-Long Gold antifade (Invitrogen, Carlsbad, CA). Actin staining was accomplished with Alexa-fluor 594-conjugated phalloidin according to manufacturer's instructions (Invitrogen).

### PCR detection of *Toxoplasma gondii*

DNA extraction from tissue samples was carried using DNeasy Blood and Tissue Kit (Qiagen Inc. Valencia, CA) following the manufacturer's instructions. Real-time PCR was performed targeting the highly conserved 35-fold-repetitive B1 gene in *T. gondii* [68]. A 25 µl-reaction mixture was prepared using 2X Power SYBR Green PCR Master Mix (Applied Biosystems, Carlsbad, CA) with 0.3 µM forward primer 5′-GGA-GGA-CTG-GCA-ACC-TGG-TGT-CG-3′ and reverse primer 5′-TTG-TTT-CAC-CCG-GAC-CGT-TTA-GCA-G-3′ [69]. Reactions were carried out in an Applied Biosystems 7500 Fast Real Time PCR System with the following thermal cycling conditions: 50°C for 2 min, 95°C for 10 min, followed by 40 cycles at 95°C for 15 sec and 60°C for 1 min. All PCR amplifications were subjected to dissociation analysis to confirm the specificity of the reaction. Quantification of parasites was accomplished by using a standard curve constructed with a series of known quantity of the *T. gondii* RH strain over a range of 6 logs corresponding to 10^5^ to 1 parasite per reaction. Uninfected tissues were used as negative controls and distilled sterile water used as zero template controls.

Parasites per cell were determined by qPCR for detection of the parasite B1 gene and host argininosuccinate lyase (NM_133768), a gene that is absent from the *Toxoplasma* genome. Aliquots of 10^6^ cells were centrifuged to pellet cells and extracellular parasites. DNA was extracted with the E.Z.N.A tissue DNA kit (Omega Bio-Tek). Using the Syber Green PerfeCTa low ROX kit (Quanta Biosciences) B1 primers (listed above) were used to determine parasite number and argininosuccinate lyase forward (5′TCT-TCG-TTA-GCT-GGC-AAC-TCA-CCT-3′) and reverse (5′ATG-ACC-CAG-CAG-CTA-AGC-AGA-TCA-3′) primers were used to determine mouse cell number in replicate samples. Standard curves were constructed as described above for both species.

### Quantitative reverse transcriptase PCR of *Arg-1* gene

Total RNA was prepared from brain tissue by Trizol purification and 1 µg was converted to cDNA using a commercially available method (Quanta Biosciences, Gaithersburg, MD). Quantitative PCR was performed on the *Arg1* gene and normalized to the expression of the housekeeping gene *GAPDH* utilizing the SYBR green method (Quanta biosciences, Gaithersburg, MD) and ABI 7500 fast machine (Life Technologies Corporation, Carlsbad, CA). Expression relative to noninfected control samples was calculated utilizing the ΔΔCt method. The primer sequences employed were: 5′-AAG-AAT-GGA-AGA-GTC-AGT-GTG-G-3′ (*Arg1* forward); 5′-GGG-AGT-GTT-GAT-GTC-AGT-GTG-3′ (*Arg1* reverse); 5′-AAT-GGT-GAA-GGT-CGG-TGT-G-3′ (*GAPDH* forward); 5′-GTG-GAG-TCA-TAC-TGG-AAC-ATG-TAG-3′ (*GAPDH* reverse).

### Cytokine ELISA and NO assay

IL-12(p40) was measured by ELISA as described [15], and TNF-α and IFN-γ was measured using a commercial kit according to the manufacturer's instructions (eBiosciences). Production of nitric oxide was detected by measurement of NO_2_/NO_3_ by the Griess reaction as described [70].

### Statistical analysis

Student's *t*-test was used to analyze statistical differences between groups. Values for *P*<0.05 were considered significant. All experiments were repeated a minimum of two times.

## Supporting Information

Figure S1 **Analysis of JAK involvement in parasite-induced STAT3 activation.** **(**A) Bone marrow-derived MØ were infected with RH strain parasites and 20 min later lysates were prepared and subjected to immunoprecipitation with anti-phosphotyrosine antibody followed by Western blotting employing antibodies specific for STAT3, JAK1 or JAK2. (B) Bone marrow-derived MØ from wild-type, *JAK3^+/+^* or *Tyk2^+/+^* mice were infected with RH strain tachyzoites (3∶1 ratio of parasites to cells), then at the indicated time points (min) cell lysates were prepared and subject to immunoblot analysis. (C) Bone marrow-derived MØ were infected as in (B) in the presence of JAK inhibitor I (80, 20, and 1 nM) or the equivalent dilution of DMSO carrier. Cell lysates were prepared for immunoblot analysis 30 min post infection. (D) MØ were treated with JAK2 inhibitor III and infected with parasites or treated with rIL-6 (100 ng/ml). Cell lysates were prepared 30 min later and subjected to Western blotting with antibodies specific for phosphorylated Tyr (Y) STAT3 or Erk1/2. These experiments were performed 3 times with similar results.
(TIF)

Figure S2 **Targeted gene replacement at the** ***ROP16*** **locus.** (A) Strategy for disruption of the *ROP16* gene by a double-crossover homologous recombination event in the strain RH*Δku80Δhxgprt* by using a ∼1.2 kb 5′ target flank and a ∼1.2 kb 3′ target flank on plasmid pΔROP16. The PCR strategy for genotype verification is depicted using primer pairs to assay for products from the PCR (not to scale). (B, C, D) A panel of six MPA resistant clones (numbered 1 to 6) was evaluated in PCR assays to validated patterns consistent with ROP16 knockout. Lane M is the DNA size ladder and lane C is the parental control strain with the ROP16 locus intact. (B) The parental strain control was positive for PCR 1 (396 bp), to assess deletion of ROP16 coding region, and the PCR 2 (586 bp) product. (C, D) The parental strain control was negative for PCR 3 (1240 bp) and PCR 4 (1218 bp) product. Targeted ΔROP16 knockouts are positive for the PCR 2, PCR 3 (5′ integration) and PCR 4 (3′ integration) products, and negative for the PCR 1 (deletion) product. All six MPA-resistant clones show a pattern consistent with a targeted deletion of a ∼2.2 kb region of the *ROP16* gene. (E) Strategy for complementation of the ΔROP16 knockout strain (RH*Δku80Δrop16::HXGPRT*) with a c-terminal HA-tagged copy the type I RH *rop16* coding region by double-crossover homologous recombination. The PCR strategy for genotype verification is depicted using primer pairs to assay for products from the PCR (not to scale). (F, G, H) A panel of six 6TX resistant clones (numbered 1 to 6) was evaluated in PCR assay to validate patterns consistent with ROP16 complementation. Lane M is the DNA size ladder, lane C is the RH*Δku80Δhxgprt* control strain (ROP16 intact) and C* is the parental ΔROP16 knockout control strain lacking ROP16. (F) The RH*Δku80Δhxgprt* control strain and all 6TX resistant clones were positive for PCR 1 (396 bp). (G) (5′ integration) The RH*Δku80Δhxgprt* control strain and all 6TX resistant clones were positive for PCR 5 (1719 bp). The ΔROP16 knockout strain (lane C*) was negative. (H) (3′ integration) The RH*Δku80Δhxgprt* control strain and all 6TX resistant clones were positive for PCR 6 (1078 bp). The ΔROP16 knockout strain (lane C*) was negative. All six 6TX-resistant clones show a pattern consistent with insertion of a single copy of the type I ROP16 coding region at the ROP16 locus.
(TIF)

Figure S3 **Deletion of ROP16 does not alter serine phosphorylation of STAT3.** Bone marrow-derived MØ were infected with RH or ΔROP16 tachyzoites (3: 1 ratio of parasites to cells), then total lysates were prepared at the indicated times (hr). Immunoblot analysis was carried out using antibody to phospho-Tyr^705^ and phospho-Ser^727^ STAT3. The experiment was repeated three times with similar results.
(TIF)

Figure S4 **STAT6 activation is wholly dependent on ROP16 but STAT3 activation is partially dependent on ROP16.** Bone marrow-derived MØ were infected with RH and ΔROP16 tachyzoites (3: 1 ratio of parasites to cells), then nuclear lysates were prepared at the indicated times (hr). Immunoblotting was carried out using anti-phospho-STAT6, then the blot was successively stripped and re-probed with antibody specific for total STAT6, phospho-STAT3, total STAT and PARP.
(TIF)

Figure S5 **In vivo activation of STAT3 and STAT6 during intraperitoneal infection.** Mice (C57BL/6 strain) were inoculated with 10^6^ RH, ΔROP16 and ΔROP16:1 tachyzoites and peritoneal exudate cells were collected 5 days later. Total cell lysates were immunoblotted with antibody to phospho-STAT3, then blots were successively stripped and re-probed for total STAT3, phospho-STAT-6 and total STAT6. Each lane represents a single mouse.
(TIF)

Figure S6 **Mice infected with RH and ΔROP16 tachyzoites display equivalent mortality.** Mice (CF1 strain) were infected with 100 tachyzoites of each strain by i. p. inoculation.
(TIF)

Figure S7 **Increased infection in the peritoneal cavity by ΔROP16 tachyzoites.** A, Mice were infected with 10^6^ RH or **Δ**ROP16 parasites, then peritoneal exudate cells were collected at the indicated times post-inoculation. Cells were stained with anti-CD11b and anti-*Toxoplasma* SAG-1 and subsequently analyzed by flow cytometry. This experiment is representative of three performed. B, Mice (n = 5 per group) were infected with 10^5^ RH and ΔROP16 tachyzoites and cells in the peritoneal cavity were collected 72 hr for qPCR analysis of the *Toxoplasma* B1 gene relative to host arginosuccinate lyase. The data are expressed as parasites per peritoneal exudate cell. Each symbol represents an individual mouse. *, p<0.01. The experiment was repeated twice with similar results.
(TIF)

Table S1 **Primers used in ROP16 knockout and complementation construction and validation.** Bold segments correspond to *T. gondii* sequences in primers used for fusing gene segments.
(DOC)
