# Supplementary figures and images for: Toxoplasma gondii Rhoptry Kinase ROP16 Activates STAT3 and STAT6 Resulting in Cytokine Inhibition and Arginase-1-Dependent Growth Control
Source: PLoS Pathog. 2011 Sep 8;7(9):e1002236. doi: 10.1371/journal.ppat.1002236 (PMC3169547; doi:10.1371/journal.ppat.1002236)

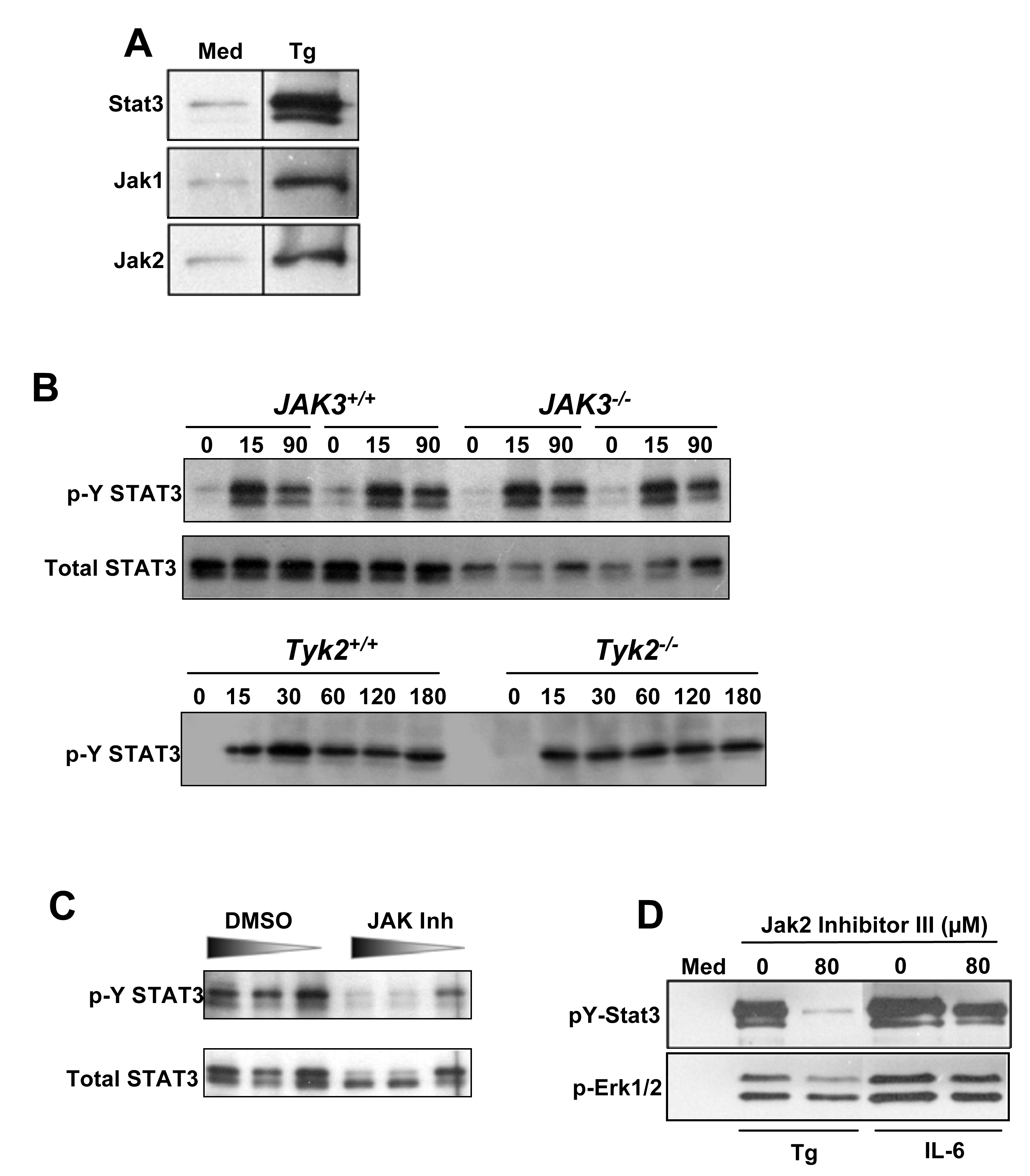

Supplement: Figure S1 — Analysis of JAK involvement in parasite-induced STAT3 activation. (A) Bone marrow-derived MØ were infected with RH strain parasites and 20 min later lysates were prepared and subjected to immunoprecipitation with anti-phosphotyrosine antibody followed by Western blotting employing antibodies specific for STAT3, JAK1 or JAK2. (B) Bone marrow-derived MØ from wild-type, JAK3+/+ or Tyk2+/+ mice were infected with RH strain tachyzoites (3∶1 ratio of parasites to cells), then at the indicated time points (min) cell lysates were prepared and subject to immunoblot analysis. (C) Bone marrow-derived MØ were infected as in (B) in the presence of JAK inhibitor I (80, 20, and 1 nM) or the equivalent dilution of DMSO carrier. Cell lysates were prepared for immunoblot analysis 30 min post infection. (D) MØ were treated with JAK2 inhibitor III and infected with parasites or treated with rIL-6 (100 ng/ml). Cell lysates were prepared 30 min later and subjected to Western blotting with antibodies specific for phosphorylated Tyr (Y) STAT3 or Erk1/2. These experiments were performed 3 times with similar results. (TIF) [file ppat.1002236.s001.tif]

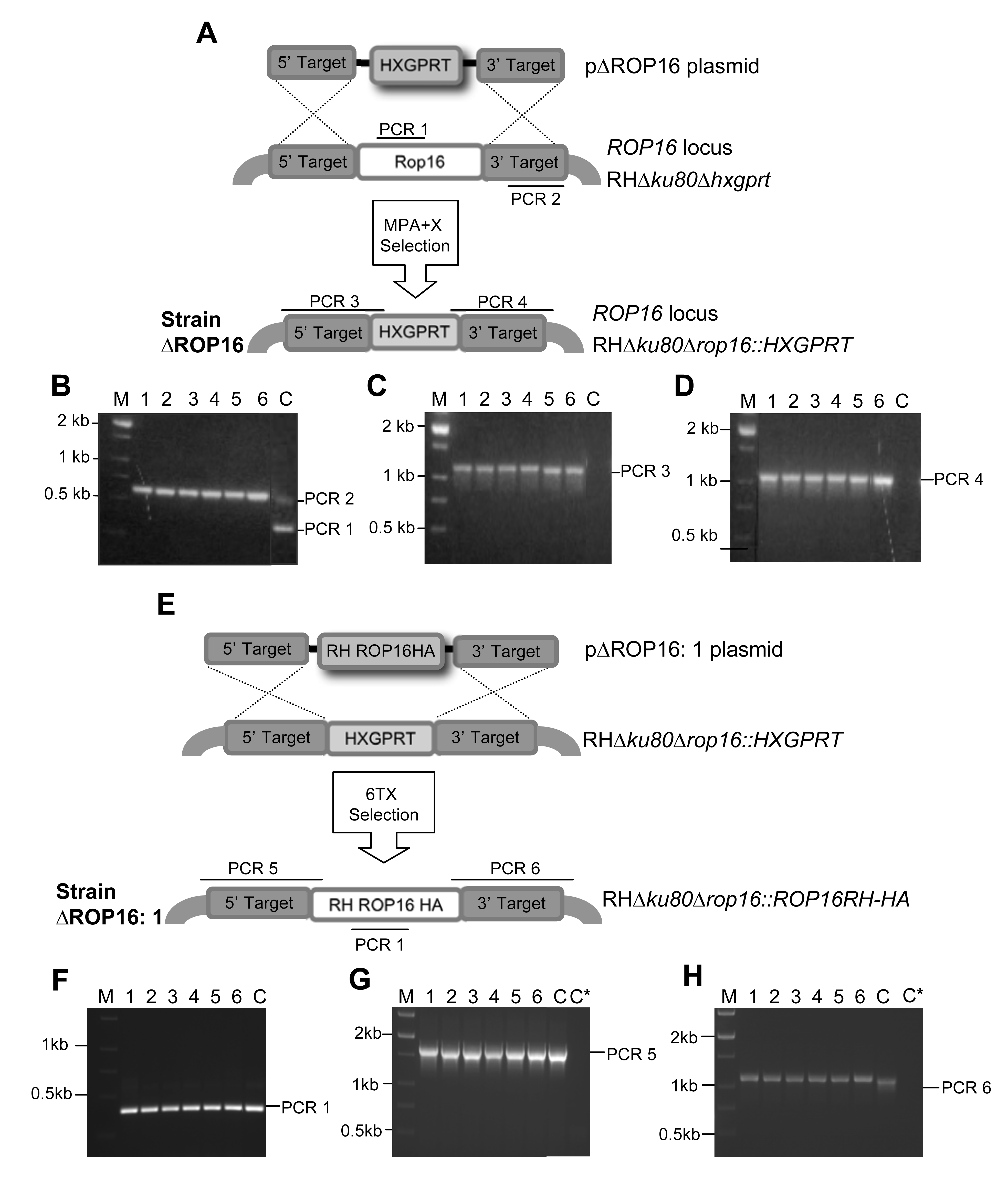

Supplement: Figure S2 — Targeted gene replacement at the ROP16 locus. (A) Strategy for disruption of the ROP16 gene by a double-crossover homologous recombination event in the strain RHΔku80Δhxgprt by using a ∼1.2 kb 5′ target flank and a ∼1.2 kb 3′ target flank on plasmid pΔROP16. The PCR strategy for genotype verification is depicted using primer pairs to assay for products from the PCR (not to scale). (B, C, D) A panel of six MPA resistant clones (numbered 1 to 6) was evaluated in PCR assays to validated patterns consistent with ROP16 knockout. Lane M is the DNA size ladder and lane C is the parental control strain with the ROP16 locus intact. (B) The parental strain control was positive for PCR 1 (396 bp), to assess deletion of ROP16 coding region, and the PCR 2 (586 bp) product. (C, D) The parental strain control was negative for PCR 3 (1240 bp) and PCR 4 (1218 bp) product. Targeted ΔROP16 knockouts are positive for the PCR 2, PCR 3 (5′ integration) and PCR 4 (3′ integration) products, and negative for the PCR 1 (deletion) product. All six MPA-resistant clones show a pattern consistent with a targeted deletion of a ∼2.2 kb region of the ROP16 gene. (E) Strategy for complementation of the ΔROP16 knockout strain (RHΔku80Δrop16::HXGPRT) with a c-terminal HA-tagged copy the type I RH rop16 coding region by double-crossover homologous recombination. The PCR strategy for genotype verification is depicted using primer pairs to assay for products from the PCR (not to scale). (F, G, H) A panel of six 6TX resistant clones (numbered 1 to 6) was evaluated in PCR assay to validate patterns consistent with ROP16 complementation. Lane M is the DNA size ladder, lane C is the RHΔku80Δhxgprt control strain (ROP16 intact) and C* is the parental ΔROP16 knockout control strain lacking ROP16. (F) The RHΔku80Δhxgprt control strain and all 6TX resistant clones were positive for PCR 1 (396 bp). (G) (5′ integration) The RHΔku80Δhxgprt control strain and all 6TX resistant clones were positive for PCR 5 (1719 bp [file ppat.1002236.s002.tif]

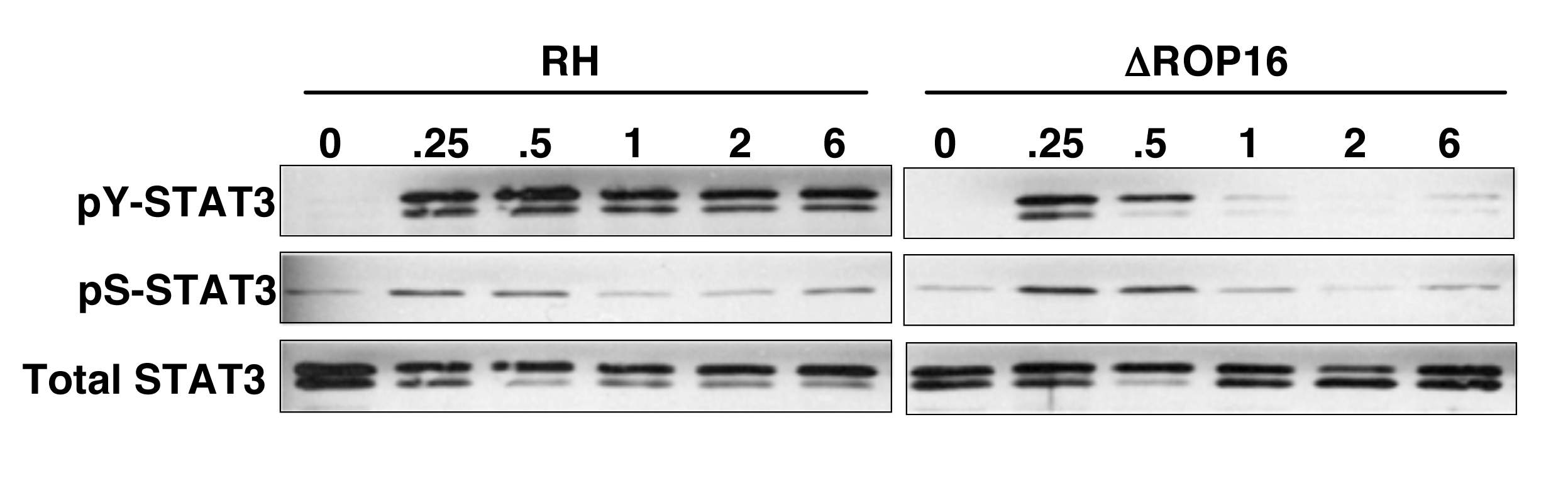

Supplement: Figure S3 — Deletion of ROP16 does not alter serine phosphorylation of STAT3. Bone marrow-derived MØ were infected with RH or ΔROP16 tachyzoites (3: 1 ratio of parasites to cells), then total lysates were prepared at the indicated times (hr). Immunoblot analysis was carried out using antibody to phospho-Tyr705 and phospho-Ser727 STAT3. The experiment was repeated three times with similar results. (TIF) [file ppat.1002236.s003.tif]

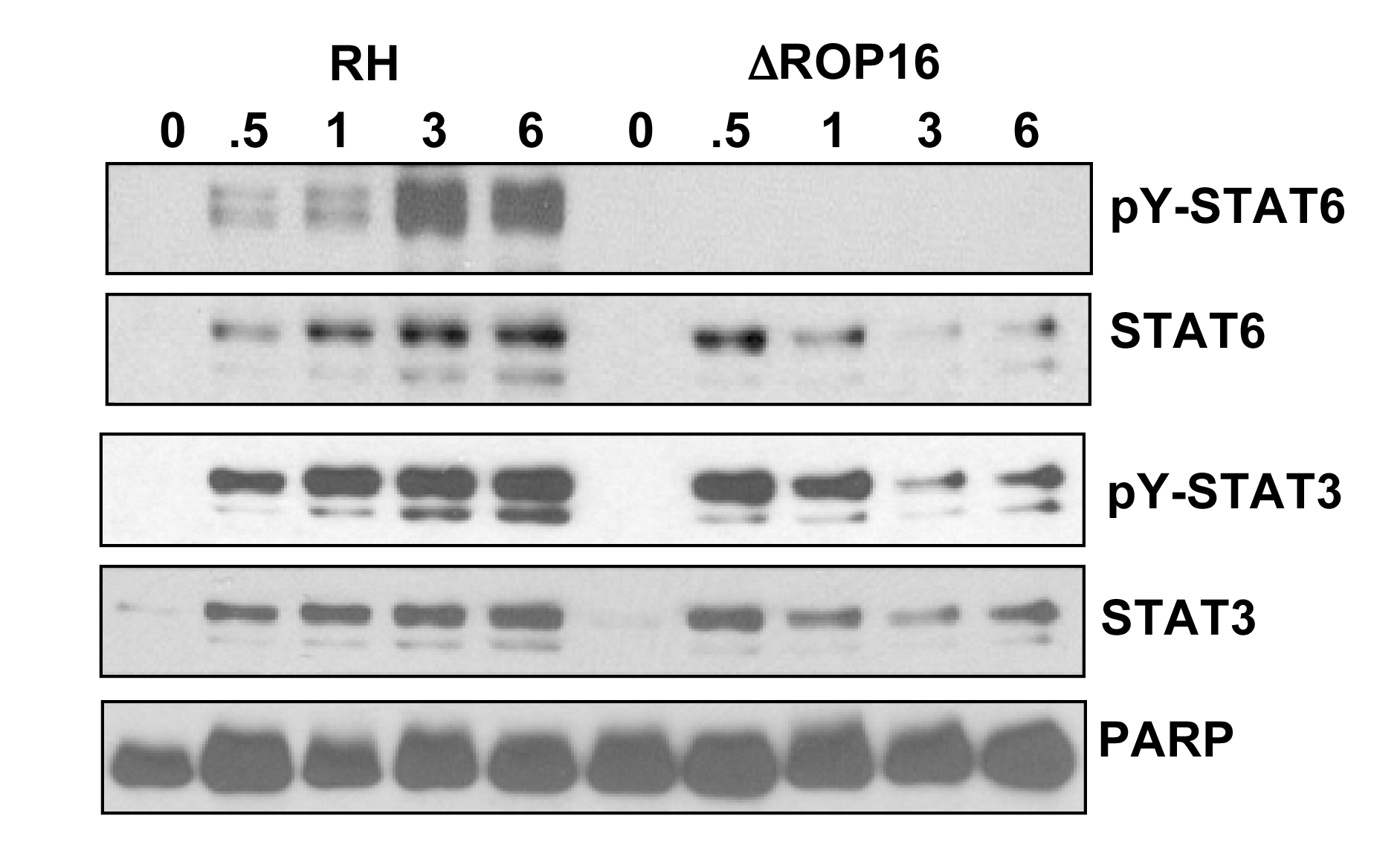

Supplement: Figure S4 — STAT6 activation is wholly dependent on ROP16 but STAT3 activation is partially dependent on ROP16. Bone marrow-derived MØ were infected with RH and ΔROP16 tachyzoites (3: 1 ratio of parasites to cells), then nuclear lysates were prepared at the indicated times (hr). Immunoblotting was carried out using anti-phospho-STAT6, then the blot was successively stripped and re-probed with antibody specific for total STAT6, phospho-STAT3, total STAT and PARP. (TIF) [file ppat.1002236.s004.tif]

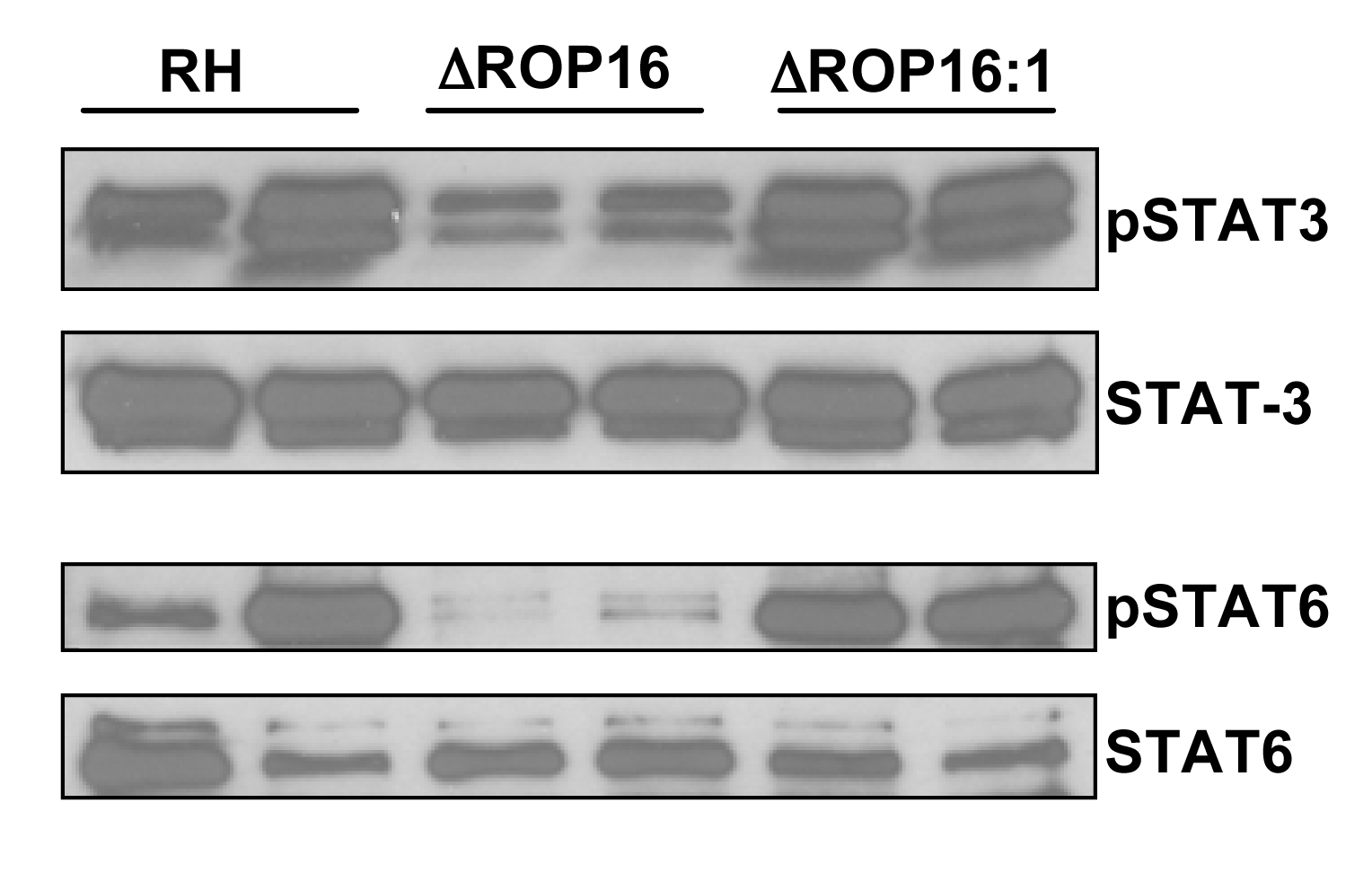

Supplement: Figure S5 — In vivo activation of STAT3 and STAT6 during intraperitoneal infection. Mice (C57BL/6 strain) were inoculated with 106 RH, ΔROP16 and ΔROP16:1 tachyzoites and peritoneal exudate cells were collected 5 days later. Total cell lysates were immunoblotted with antibody to phospho-STAT3, then blots were successively stripped and re-probed for total STAT3, phospho-STAT-6 and total STAT6. Each lane represents a single mouse. (TIF) [file ppat.1002236.s005.tif]

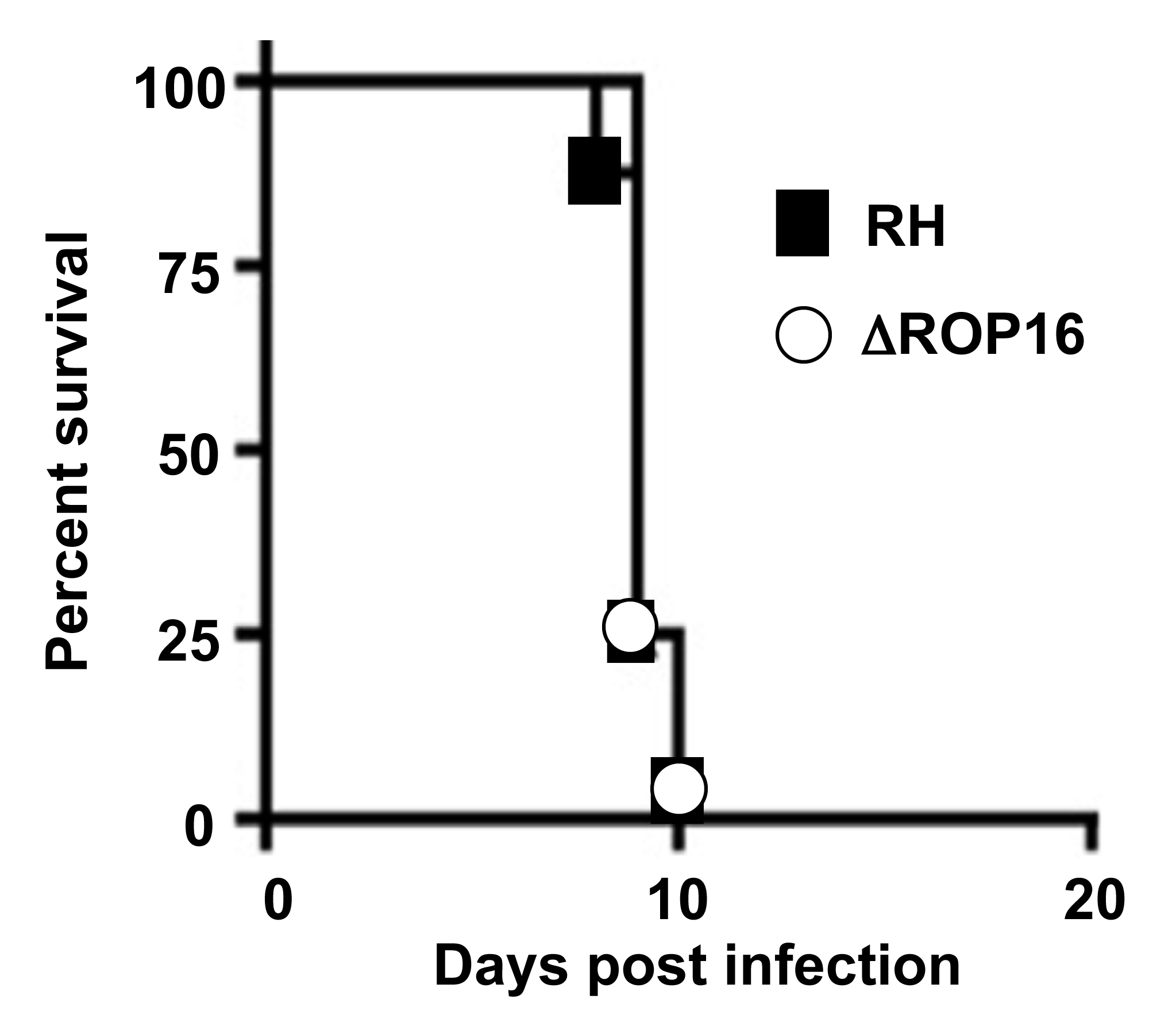

Supplement: Figure S6 — Mice infected with RH and ΔROP16 tachyzoites display equivalent mortality. Mice (CF1 strain) were infected with 100 tachyzoites of each strain by i. p. inoculation. (TIF) [file ppat.1002236.s006.tif]

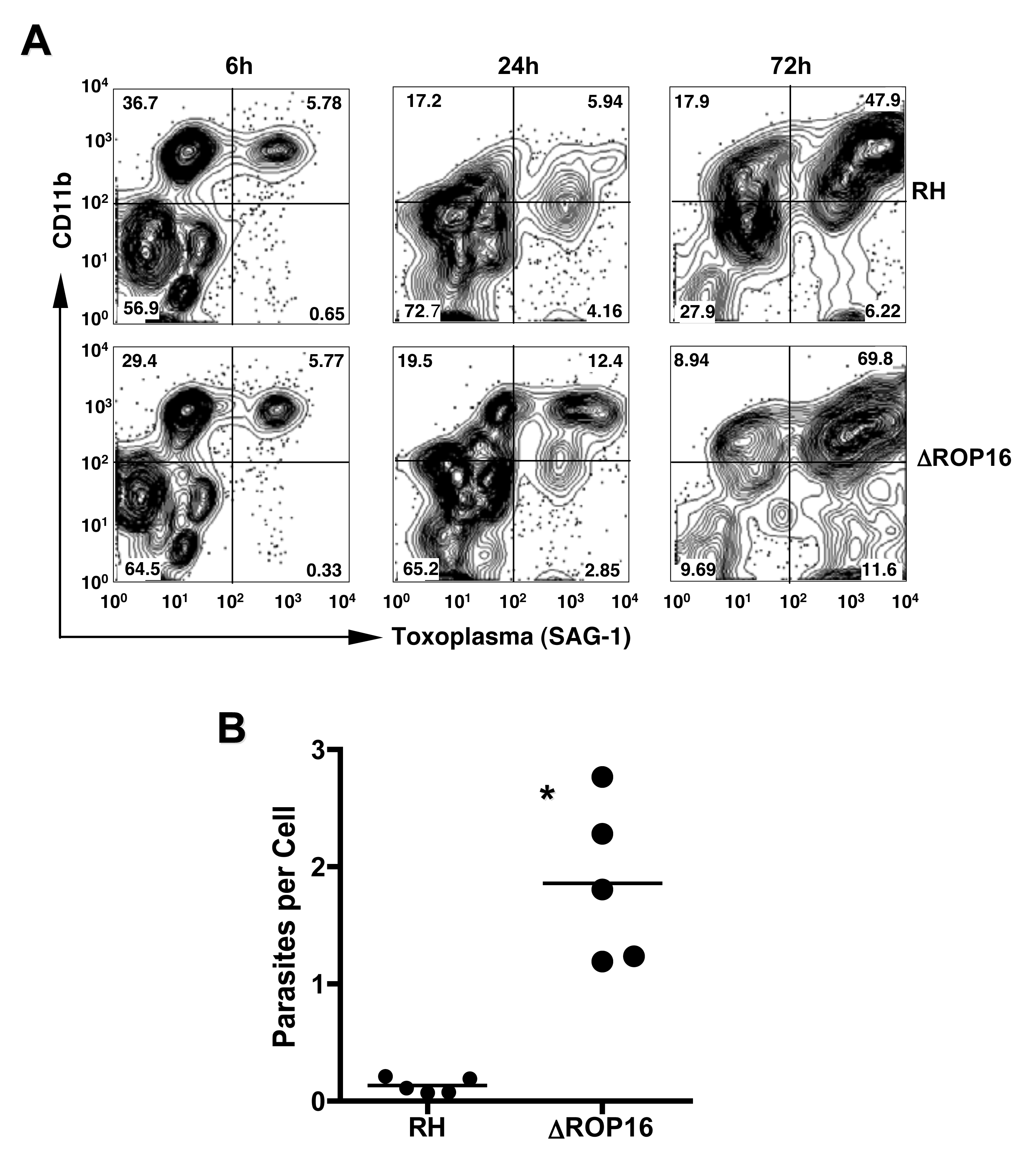

Supplement: Figure S7 — Increased infection in the peritoneal cavity by ΔROP16 tachyzoites. A, Mice were infected with 106 RH or ΔROP16 parasites, then peritoneal exudate cells were collected at the indicated times post-inoculation. Cells were stained with anti-CD11b and anti-Toxoplasma SAG-1 and subsequently analyzed by flow cytometry. This experiment is representative of three performed. B, Mice (n = 5 per group) were infected with 105 RH and ΔROP16 tachyzoites and cells in the peritoneal cavity were collected 72 hr for qPCR analysis of the Toxoplasma B1 gene relative to host arginosuccinate lyase. The data are expressed as parasites per peritoneal exudate cell. Each symbol represents an individual mouse. *, p<0.01. The experiment was repeated twice with similar results. (TIF) [file ppat.1002236.s007.tif]
