# Supplementary material for: Toxoplasma gondii Rhoptry Kinase ROP16 Activates STAT3 and STAT6 Resulting in Cytokine Inhibition and Arginase-1-Dependent Growth Control
Source: PLoS Pathog. 2011 Sep 8;7(9):e1002236. doi: 10.1371/journal.ppat.1002236 (PMC3169547; doi:10.1371/journal.ppat.1002236)
Supplement: Table S1 — Primers used in ROP16 knockout and complementation construction and validation. Bold segments correspond to T. gondii sequences in primers used for fusing gene segments. (DOC) [file ppat.1002236.s008.doc]

**Table S1.** Primers used in ROP16 knockout and complementation construction and validation

| Primer | Sequence |
| --- | --- |
| ROP16 F1 | TTGGGTAACGCCAGGGTTTTCCCAGTCACGACGGTTTAAAC**GGCGTTCTGTGTTAGCTGCCAG** |
| ROP16 R1 | GCGGGTTTGAATGCAAGGTTTCGTGCTGATCAAACTAGT**CATAGGCACTACCAGTGGTGCATTG** |
| ROP16 F2 | TTCTGGCAGGCTACAGTGACACCGCGGTGGAGGACTAGT**CGAATCTGATCCAGCAGTGATGGG** |
| ROP16 R2 | GTGAGCGGATAACAATTTCACACAGGAAACAGCGCGGCCGC**CCCATGTCTCTTAAGGTGTGCGTC** |
| ROP16 CXF | GCAACTACTTCGACGGAACCGTC |
| ROP16 CXR | CCTTAACAGGCAAATGAACACGAGCT |
| ROP16 EXF | GAACTGGTTGAGAGGCTAGGAGCA |
| ROP16 ΔF | TCCTGGTCAGAGCGCTACATTGG |
| ROP16 ΔR | GGAAACACTTCGTCAACAGCTGACTC |
| ROP16 cF2.HA | TACCCATACGATGTTCCAGATTACGCT**TAGGGTGTAAGGTTCCCACCTTAACACC** |
| ROP16 cR1 | AAGCAAGCCCTTTCGTGGTCAC |
| ROP16 cF | AACTGCCTGTGATCTTGTTTGTCGC |
| ROP16 cR.HA | AGCTGTAATCTGGAACATCGTATGGGTA**CATCCGATGTGAAGAAAGTTCGGTAGTT** |
| ROP16 cvF | CGAATGTGATCCAGCAGTGATGGG |
| ROP16 cvR | GTAGTGTGGATTGAGAAGGTTGCCC |
| pminiHXF | GATAAGCTTGATCAGCACGAAACCTTG |
| pminiHXR | CCGCTCTAGAACTAGTGGATCCC |
